# Disordered metasurface enabled single-shot full-Stokes polarization imaging leveraging weak dichroism

**DOI:** 10.1038/s41467-023-42944-6

**Published:** 2023-11-07

**Authors:** Qingbin Fan, Weizhu Xu, Xuemei Hu, Wenqi Zhu, Tao Yue, Feng Yan, Peicheng Lin, Lu Chen, Junyeob Song, Henri J. Lezec, Amit Agrawal, Yanqing Lu, Ting Xu

**Affiliations:** 1 National Laboratory of Solid-State Microstructures and Collaborative Innovation Center of Advanced Microstructures, Nanjing University, Nanjing, 210093 China; 2 School of Electronic Sciences and Engineering, Nanjing University, Nanjing, 210093 China; 3 College of Engineering and Applied Sciences and Jiangsu Key Laboratory of Artificial Functional Materials, Nanjing University, Nanjing, 210093 China; 4 Physical Measurement Laboratory, National Institute of Standards and Technology, Gaithersburg, Maryland 20899 USA; 5 Maryland NanoCenter, University of Maryland, College Park, Maryland 20899 USA

**Keywords:** Imaging and sensing, Metamaterials

## Abstract

Polarization, one of the fundamental properties of light, is critical for certain imaging applications because it captures information from the scene that cannot directly be recorded by traditional intensity cameras. Currently, mainstream approaches for polarization imaging rely on strong dichroism of birefringent crystals or artificially fabricated structures that exhibit a high diattenuation typically exceeding 99%, which corresponds to a polarization extinction ratio (PER) >~100. This not only limits the transmission efficiency of light, but also makes them either offer narrow operational bandwidth or be non-responsive to the circular polarization. Here, we demonstrate a single-shot full-Stokes polarization camera incorporating a disordered metasurface array with weak dichroism. The diattenuation of the metasurface array is ~65%, which corresponds to a PER of ~2. Within the framework of compressed sensing, the proposed disordered metasurface array serves as an efficient sensing matrix. By incorporating a mask-aware reconstruction algorithm, the signal can be accurately recovered with a high probability. In our experiments, the proposed approach exhibits high-accuracy full-Stokes polarimetry and high-resolution real-time polarization imaging. Our demonstration highlights the potential of combining meta-optics with reconstruction algorithms as a promising approach for advanced imaging applications.

## Abstract (web summary)

Polarization imaging can capture information from the scene that cannot directly be recorded by traditional intensity cameras. Here, authors demonstrate a single-shot full Stokes polarization camera incorporating a disordered metasurface array with weak dichroism.

## Introduction

Polarization, a fundamental characteristic of light, plays a critical role in almost all areas of optical science and technology. For example, polarization imaging can reveal indiscernible information that cannot easily be detected with traditional intensity cameras. Examples include measuring the vector distribution of structured light beams, the texture and stress of reflective or transmissive surfaces, and/or the optical activity of biomaterials^1–3^. Conventional techniques for polarization imaging rely on strong dichroism of birefringent crystals or artificial structures, such as the use of polarization splitters or wire grid polarizers. Depending on the principle of operation, these technologies can be categorized into three types: division-of-time (DoT), division-of-amplitude (DoA) and division-of-focal-plane (DoFP). DoT devices rely on mechanically rotating the polarization dichroism elements to capture different polarization images sequentially in time, thus requiring complex mechanical control which sacrifices the temporal resolution^3^. DoA method makes measurements by splitting the light into different optical paths, each with distinct polarization optics, and using a separate focal-plane array to capture images for each path. While this method enables the simultaneous detection of multiple polarization states, it inherently requires a large propagation space to effectively segregate the split light and accomplish precise registration^4^. By leveraging a multiplexed spatial arrangement of polarization dichroism elements on top of a sensor, DoFP method not only enables simultaneous acquisition of multiple polarization images but also contributes to the integration of the imaging system^5–9^. However, compared with DoT devices, the image resolution of DoFP devices is limited because the imaging sensor is spatially sub-divided into different regions for capturing different polarization states. In addition, most traditional polarization cameras offer no response to an input circular polarization state.

Recently, by leveraging the ability of metasurfaces to provide unprecedented multifunctional control of light^10–25^, ultrathin polarization optics have been developed as a key component for realization of compact polarimeters^26–36^. Ordinary sensors cascaded with these meta-optics are capable of detecting arbitrary polarization states (linear, circular or elliptical), resulting in the measurement of full-Stokes parameters. Furthermore, based on polarization-dependent metasurface gratings, single-shot full-Stokes polarization cameras have also been demonstrated^37,38^. These demonstrations belong to the category of DoA mentioned above, and thus inevitably inherit its disadvantages. Moreover, these metasurface-based polarization elements always operate within a narrow bandwidth limited by the inherent phase dispersion. Therefore, despite significant advances in polarization measurement and imaging, these approaches still suffer from a number of fundamental limitations.

In this work, we present a novel platform based on disordered metasurface array for realizing a single-shot full-Stokes polarization camera. In contrast to previously reported devices that employ strong dichroism elements with diattenuation exceeding 99% and corresponding to a polarization extinction ratio (PER) >~100^3–8^, we design a disordered metasurface array with weak dichroism. The average diattenuation of the metasurface array is ~65%, which corresponds to a PER of ~2, allowing it to efficiently respond to various input polarization states. Generally speaking, weak dichroism will reduce the accuracy of polarization measurements and decrease the contrast in polarization imaging. Here we demonstrate that, compared with strong dichroism elements, weak dichroism meta-optics can still be used to obtain full-Stokes information with measurement accuracy comparable to other commercial polarimeters. Within the framework of compressed sensing theory, the proposed disordered metasurface array serves as an efficient compressive sampling matrix in both spatial and polarization dimensions, capable of encoding natural scenes in a sparse transform domain. From the encoded data, the corresponding mask-aware reconstruction algorithms are able to accurately recover the signal with a high probability. In contrast to metasurface grating-based polarization cameras that only use a portion of the sensor area for imaging due to the spatially separated diffraction orders^37,38^, the proposed polarization imaging system could fully utilize the sensor area and reconstruct full-Stokes polarization images. Moreover, the proposed approach is fully compatible with the architecture of conventional cameras, and thus provides a fast, simple and compact way to capture polarization information from a scene of interest.

## Results

### Disordered metasurface array

**Fig. 1:**
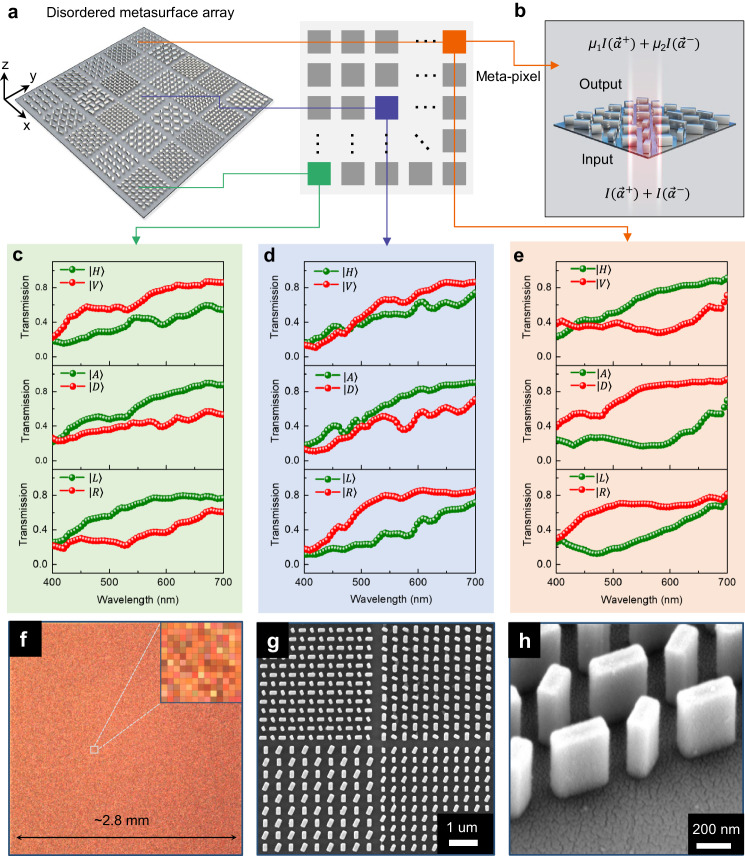
Disordered dielectric metasurface array with weak dichroism. **a** Metasurface element composed of a two-dimensional array of polarization-dependent meta-pixels. **b** The operational principle of a single meta-pixel. Each meta-pixel mapping to the sensor pixels is sensitive to two arbitrary orthogonal SoPs [${\mathop{\alpha }\limits^{ \rightharpoonup }}^{+}$,${\mathop{\alpha }\limits^{ \rightharpoonup }}^{-}$], including linear and circular polarizations. *I* represents the light intensity. ${\mu }_{1}$ and ${\mu }_{2}$ denote the transmission coefficients. **c**–**e** Experimentally measured transmission of three representative meta-pixels with operation wavelengths covering the entire visible range from 400 nm to 700 nm. **f** Optical microscope image of the fabricated metasurface device. The inset shows a zoomed-in meta-pixel array. **g**, **h** The SEM images show the top view and oblique view of the TiO_2_ nanopillars.

Within the compressed sensing theory, the Restricted Isometry Property (RIP) condition is a necessary and sufficient condition for the effective reconstruction of sparse and compressible signals^39^. It has been theoretically proven that random sampling matrices can satisfy the RIP condition, enabling them to serve as efficient sensing matrices for achieving high-accuracy signal reconstruction^40,41^. Therefore, our goal is to design a random polarization sensing matrix by utilizing an all-dielectric metasurface array. Figure 1a shows the schematic diagram of the proposed disordered metasurface array consisting of 400 × 400 meta-pixels. There are 256 different types of meta-pixels that form the metasurface array where each meta-pixel contains a periodic array of two types of anisotropic nanopillars with varying in-plane orientation and size (schematic illustration of one meta-pixel is shown in Fig. 1b). For any pair of orthogonal states of polarization (SoP), each meta-pixel is designed to provide a polarization-dependent transmission response. It should be noted that, in contrast with conventional disordered medium, here the disordered metasurface array provides ‘randomness’ in polarization response across the pixelated arrangement, rather than through the statistical properties of scattered light. In this configuration, the whole metasurface array can be directly integrated on top of an image sensor such that the incident light modulated by each meta-pixel can be captured by the corresponding photodetector beneath that meta-pixel.

Each meta-pixel consists of an array of titanium dioxide (TiO_2_) nanopillars with a rectangular cross-section (height *h* = 600 nm), arranged in a square lattice. Figure 1c–e show the experimentally measured transmission spectra of three selected meta-pixels under illumination with three pairs of orthogonal SoPs, including horizontal (|*H*〉, 0°), vertical (${{{{{\rm{|}}}}}}V{{\rangle }}$, 90°), anti-diagonal (|*A*〉, 45°), diagonal (|*D*〉, 135°), left-handed circular (${{{{{\rm{|}}}}}}L{{\rangle }}$) and right-handed circular (|*R*〉) polarization states. The optical microscope images and scanning electron microscope (SEM) images of the fabricated metasurface array and meta-pixels are shown in Fig. 1f–h, respectively. As designed, the meta-pixels provide the desired polarization-dependent transmission response across the entire visible range. Compared with conventional polarization optics with strong dichroism, the dichroism of the designed meta-pixel is much weaker. For example, at a representative wavelength of 550 nm, the average diattenuation of the whole disordered metasurface array is ~65%, corresponding to a polarization extinction ratio of ~2. In addition, it is worth noting that the measured average transmission efficiency from all the meta-pixels is ~62.5% at a wavelength of 550 nm for input unpolarized light, which is higher than the theoretical limit of a polarization-filter-based (*e.g*., wire grid polarizer) imaging system. Therefore, the weak dichroism not only facilitates relatively high transmission efficiencies for both the orthogonal SoPs enhancing the signal-to-noise ratio (SNR) in imaging applications, but it also simplifies the design of nanostructures and reduces the requirements on fabrication accuracy. Details about the metasurface design are described in Supplemental Material S1.

### Operation scheme

**Fig. 2:**
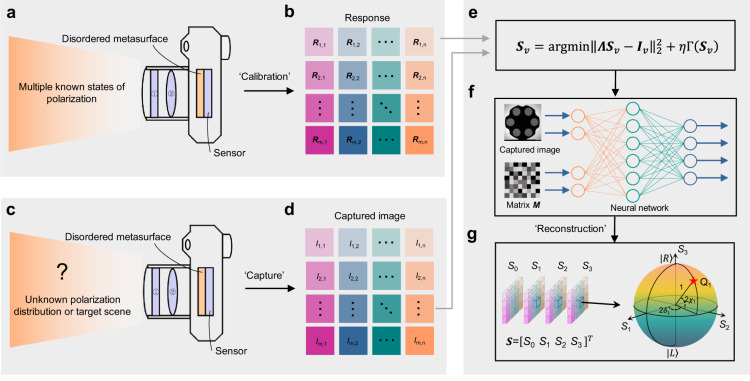
The operational principle of the nanophotonic polarization camera consists of three steps: calibration, capture, and reconstruction. **a** Schematic of the calibration process. ➀ denotes a narrowband spectral filter. ➁ denotes an imaging lens. **b** The responsivity matrix ${{{{{\boldsymbol{R}}}}}}$ of the whole meta-device is calibrated from the photoresponses to the three pairs of orthogonal SoPs of incident light. **c** Schematic of the capture process. Scattered optical waves at each position (*x*, *y*) of target scene represented an unknown SoP ${{{{{\boldsymbol{S}}}}}}={\left[{S}_{0},{S}_{1},{S}_{2},{S}_{3}\right]}^{T}$, illuminates the imaging system. **d** A single-shot capture process generates the polarization-encoded image ***I***. **e** Mathematical description of the relation between ${{{{{{\boldsymbol{S}}}}}}}_{{{{{{\boldsymbol{v}}}}}}}$, ***I***_**v**_, and ${{{{{\boldsymbol{\Lambda }}}}}}$. **f** SoP reconstruction by the convolutional neural network. **g** Reconstructed SoP and corresponding position on Poincaré sphere.

The operation scheme of the proposed nanophotonic polarization camera is illustrated in Fig. 2. It consists of three steps: (1) calibration, (2) capture, and (3) reconstruction. At the calibration stage, by measuring the transmitted light intensities of three pairs of orthogonal polarization bases in sequence, the responsivity matrix ${{{{{\boldsymbol{R}}}}}}$ is constructed (Fig. 2a, b). For each meta-pixel, the transmitted light intensity ${R}_{i,j}$ (where $i$ and $j$ represent indices in a two-dimensional meta-pixel array) and the corresponding full-Stokes vector ${{{{{\boldsymbol{S}}}}}}$ of the incident light have the following relationship:

1 $${R}_{i,j}={{{{{{\boldsymbol{M}}}}}}}_{i,j}{{{{{\boldsymbol{\cdot }}}}}}{{{{{\boldsymbol{S}}}}}},$$

where ${{{{{{\boldsymbol{M}}}}}}}_{i,j}$ denotes a Stokes-like vector which describes the polarization transmission property for that meta-pixel. With three pairs of known incident polarization bases and the corresponding responsivity matrix element ${R}_{i,j}$, the transmission vector ${{{{{{\boldsymbol{M}}}}}}}_{i,j}$ of each meta-pixel can be retrieved by solving the inverse problem of Eq. (1). The three-dimensional (3D) transmission matrix ${{{{{\boldsymbol{M}}}}}}$ of the whole disordered meta-pixel array encompasses the sampling features of all polarization analyzers.

In the next capture step (Fig. 2c and d), the photoresponse to the target scene with an unknown polarization distribution is measured through a single exposure, generating the polarization-encoded image ${{{{{\boldsymbol{I}}}}}}$. The captured image can be written as:

2 $${{{{{{\boldsymbol{I}}}}}}}_{v}={{{{{\boldsymbol{\Lambda }}}}}}{{{{{{\boldsymbol{S}}}}}}}_{v}+{{{{{\boldsymbol{N}}}}}},$$

where ${{{{{{\boldsymbol{I}}}}}}}_{v}$ is the vectorized form of ${{{{{\boldsymbol{I}}}}}}$, ${{{{{\boldsymbol{\Lambda }}}}}}={{{{{\rm{diag}}}}}}({{{{{{\boldsymbol{M}}}}}}}_{{{{{\mathrm{1,1}}}}}}^{T},\,{{{{{{\boldsymbol{M}}}}}}}_{{{{{\mathrm{1,2}}}}}}^{T},\ldots,\,{{{{{{\boldsymbol{M}}}}}}}_{m,n}^{T})$ is the polarization transmission matrix in diagonal form, ${{{{{{\boldsymbol{S}}}}}}}_{v}={[{{{{{{\boldsymbol{S}}}}}}}_{{{{{\mathrm{1,1}}}}}}^{T},\,{{{{{{\boldsymbol{S}}}}}}}_{{{{{\mathrm{1,2}}}}}}^{T},\ldots,\,{{{{{{\boldsymbol{S}}}}}}}_{m,n}^{T}]}^{T}$ denotes the target full-Stokes polarization image in vectorized form and ${{{{{\boldsymbol{N}}}}}}$ denotes the noise. Therefore, the SoP of the incident light can be reconstructed by solving the following problem (Fig. 2e):

3 $${{{{{{\boldsymbol{S}}}}}}}_{v}={{{{{\rm{argmin}}}}}}||{{{{{\boldsymbol{\Lambda }}}}}}{{{{{{\boldsymbol{S}}}}}}}_{v}-{{{{{{\boldsymbol{I}}}}}}}_{v}|{|}_{2}^{2}+\eta \Gamma ({{{{{{\boldsymbol{S}}}}}}}_{v}).$$

Here, the symbol “$\Gamma$” is a sparsity regularization operator that is determined by the statistical prior knowledge^42–45^. The first term denotes data fidelity and the second term denotes sparsity regularization. $\eta$ denotes the balancing coefficient of these two terms.

Since Eq. 2 is an underdetermined equation, it is only possible to obtain a unique solution through restricting the solution space to a smaller range of values. To solve this ill-posed inverse problem, we propose a mask-aware, deep compressed sensing-based reconstruction method. The reconstruction network, consisting of a fully convolutional neural network, reconstructs the full-Stokes polarization images of the object with the guidance of 3D transmission matrix ${{{{{\boldsymbol{M}}}}}}$ in an end-to-end mask-aware manner (Fig. 2f). Compared with traditional spatial interpolation methods, the large number of parameters in deep neural network (DNN) and its complex multilayer nonlinear structure grant the approximation capability to automatically extract implicit sparse representation from massive training data and memorize them in the convolutional layer weights. During inference, the trained DNN reconstructs high resolution images from measurements encoded by the sensing matrix and suppresses the measurement errors introduced in both calibration and capture processes by implicitly restoring the sparse features in the latent sparse transform domain learned from a large dataset. During training, both mean square error (MSE) and ${L}_{1}$ norm are introduced to systematically evaluate the quality of the output image. Based on this proposed approach, full-Stokes polarization images can be reconstructed in real-time (Fig. 2g). In fact, the disordered metasurface array proposed here implies a non-restricted coding strategy which introduces an easier-to-implement coding space and the mask-aware training process collaborated with the pre-calibrated device matrix also enhances the method’s tolerance to fabrication errors in metasurface devices.

To compare the performance of our metasurface array with other traditional ordered full-Stokes micro-polarizer array designs exhibiting strong dichroism, we conduct a theoretical analysis of these imaging systems. According to the compressed sensing theory^46–48^, the imaging process can be rewritten in the standard form of an underdetermined system of linear equations: ${{{{{\boldsymbol{y}}}}}}={{{{{\boldsymbol{\Lambda }}}}}}{{{{{\boldsymbol{x}}}}}}={{{{{\boldsymbol{\Lambda }}}}}}{{{{{\boldsymbol{\Psi }}}}}}{{{{{\boldsymbol{\theta }}}}}}+{{{{{\boldsymbol{N}}}}}}$, where ${{{{{\boldsymbol{\theta }}}}}}\in {{\mathbb{R}}}^{n}$ is the representation of input signal ${{{{{\boldsymbol{x}}}}}}\in {{\mathbb{R}}}^{n}$ in basis ${{{{{\boldsymbol{\Psi }}}}}}\in {{\mathbb{R}}}^{n\times n}$. If the input signal ${{{{{\boldsymbol{x}}}}}}$ is *k*-sparse on the basis ${{{{{\boldsymbol{\Psi }}}}}}$, i.e., there is only *k* non-zero elements in vector ${{{{{\boldsymbol{\theta }}}}}}$, ${{{{{\boldsymbol{\theta }}}}}}$ can be exactly recovered using ${\ell }_{1}$ minimization^49^ when the number of measurements *m* satisfies: $m\ge c{\mu }^{2}\left({{{{{\boldsymbol{\Lambda }}}}}},\,{{{{{\boldsymbol{\Psi }}}}}}\right)k\log \left(n\right)$. Here, $\mu \left({{{{{\boldsymbol{\Lambda }}}}}},\,{{{{{\boldsymbol{\Psi }}}}}}\right)=\sqrt{n}\mathop{\max }\limits_{\left(1\le i,j\le n\right)}|\langle {\varLambda }_{i},\,{\varPsi }_{j}\rangle |$, where ${\varLambda }_{i}$ represents the *i*-th row of ${{{{{\boldsymbol{\Lambda }}}}}}$, ${\varPsi }_{j}$ represents the *j*-th column of ${{{{{\boldsymbol{\Psi }}}}}}$. *c* is a known positive constant. With the sparsity prior, the solution space of the equations becomes much smaller than that in the raw spatial domain and only depends on a few number of unknown parameters in the sparse transform domain. This premise radically changes the ill-posed problem, making the search for solutions feasible. Based upon the theory, the smaller $\mu \left({{{{{\boldsymbol{\Lambda }}}}}},\,{{{{{\boldsymbol{\Psi }}}}}}\right)$, the fewer sampling points are required, allowing the signal with sparsity prior to be reconstructed at a low sampling rate. Therefore, we define the sampling efficiency as the coherence $\mu \left({{{{{\boldsymbol{\Lambda }}}}}},\,{{{{{\boldsymbol{\Psi }}}}}}\right)$ to compare the performance of DoFP systems.

**Fig. 3:**
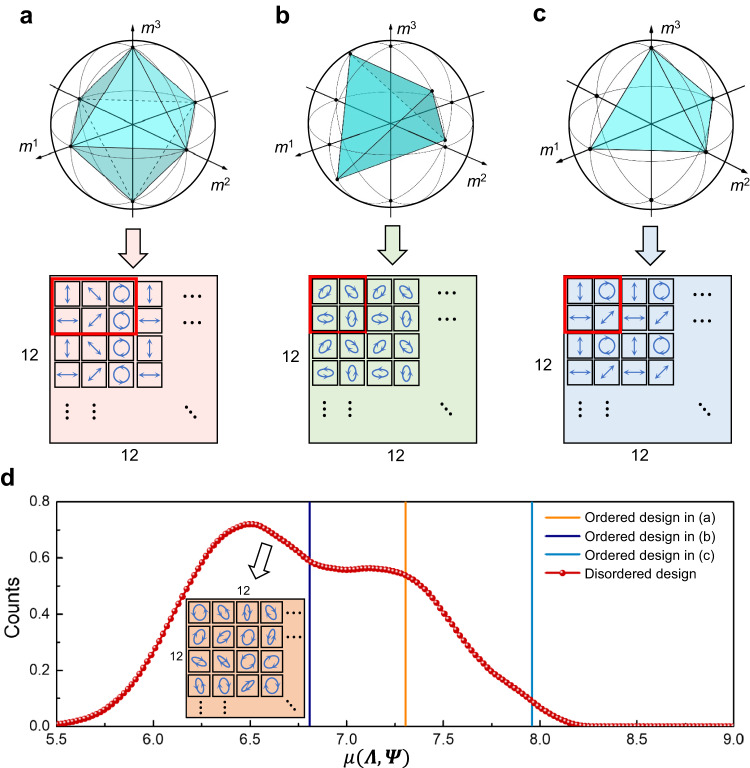
Comparison of sampling efficiency between ordered and disordered designs. **a**–**c** Ordered polarization filter arrangements and the corresponding volume enclosed within the Poincaré sphere. The red box denotes the basic arrangement unit. The coordinate axes represent the last three components of the Stokes-like vector ${{{{{{\boldsymbol{M}}}}}}}_{i,j}=[{m}_{i,j}^{0},\,{m}_{i,j}^{1},\,{m}_{i,j}^{2},\,{m}_{i,j}^{3}]^{T}$. **d** $\mu ({{{{{\boldsymbol{\Lambda }}}}}},{{{{{\boldsymbol{\Psi }}}}}})$ on different arrangements. Due to variations in sampling efficiency within each 12 × 12 patch of the designed disordered metasurface array, we randomly select 10,000 patches from our 400 × 400 metasurface array to calculate the sampling efficiency and illustrate these results in the form of density plot for fairness. The inset in (**d**) is a 12 × 12 patch, serving as an example to illustrate the arrangement of random polarization encoding.

Specifically, we compare the sampling efficiency of our disordered metasurface array with three types of conventional ordered polarization filter arrangements (Fig. 3), namely the octahedral arrangement^32^, tetrahedral arrangement^50^, and the arrangement described in^51^. The sparse representation ${{{{{\boldsymbol{\Psi }}}}}}$ is derived from the recently released full-Stokes polarization dataset^52^ utilizing the principal component analysis (PCA) method. As shown in Fig. 3d, the sampling efficiency of our disordered metasurface array is comparable to that of conventional ordered sampling schemes. This indicates the potential of the proposed disordered sampling scheme for achieving high-performance polarization measurements or polarization imaging. Compared with ordered arrangements, the disorder property of the proposed random sampling strategy could be preserved after the sparse transformation, and thus leading to a larger number of unique sampling points in the sparse transform domain, boosting the solution of the equation with higher numerical stability. Additional details about the sampling efficiency analysis are provided in the Supplemental Material S2.

### Full-Stokes polarimetric measurements

To verify the effectiveness of the proposed method, we employed a 16 × 16 subarray as a single-point polarimeter to perform polarization measurements. In experiments, a light beam emanating from a light emitting diode (LED) source equipped with a spectral filter is transmitted through a linear polarizer and a waveplate to generate an arbitrary SoP. To capture the optical response of the metasurface device, we imaged the surface of the metasurface device onto the sensor using a custom-built microscopy system. Simultaneously, the SoPs of incident beam are also monitored by using a commercial polarimeter as reference (Thorlabs PAX1000VIS).

**Fig. 4:**
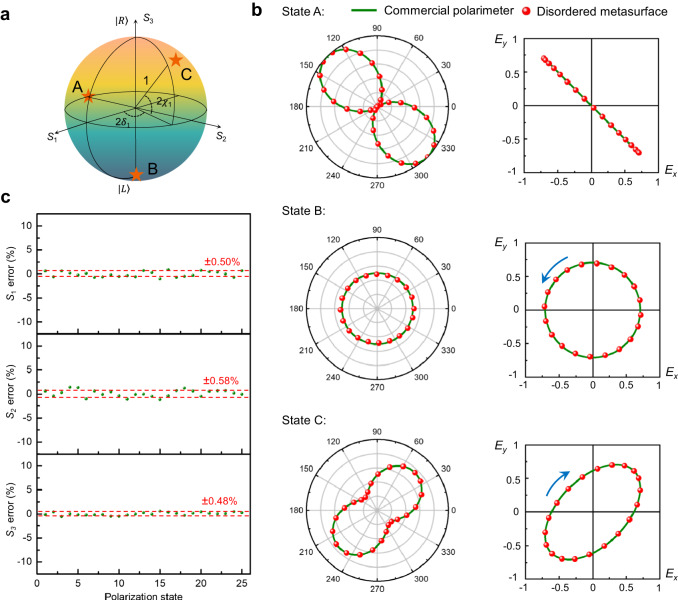
Full-Stokes polarimetric measurements. **a** Three representative SoPs are chosen for polarization measurement. **b** Comparison of the SoPs obtained using a commercial polarimeter (green solid lines) and our method (red dots), using polar plots and polarization ellipses, at an operational wavelength of 550 nm. The radius on the polar plot indicates the normalized light intensity. Blue arrows denote the handedness of light. **c** The reconstruction errors of Stokes parameters (*S*_1_, *S*_2_, *S*_3_) from 25 arbitrarily selected SoPs on Poincaré sphere.

A number of independent SoP measurements are performed at a wavelength of 550 nm. Figure 4a shows the positions of three representative SoPs on the Poincaré sphere, while Fig. 4b presents their corresponding experimental results. To present the accuracy of the reconstructed SoPs, we depict the results using both polar diagrams and polarization ellipses (Fig. 4b). As shown, the measurement results obtained with the metasurface array (red dot) exhibit near-perfect consistency with those obtained from a commercial polarimeter (green solid line). Furthermore, as shown in Fig. 4c, through the analysis of the reconstructed Stokes parameters of 25 arbitrarily selected SoPs, the average measurement errors of *S*_1_*, S*_2_, and *S*_3_ are ±0.50%, ±0.58%, and ±0.48%, respectively. The degree of polarization (DoP, given by $\sqrt{{S}_{1}^{2}+{S}_{2}^{2}+{S}_{3}^{2}}/{S}_{0}$), is the ratio of polarized light to total radiance. The reconstruction error for DoP is ±0.58%. Compared with other metasurface-based polarimetric detection techniques, our method exhibits higher performance in measurement accuracy (summarized in Supplementary Material Table S1). In our method, accurate polarization measurements are achieved by pre-calibrating the transmission function of disordered metasurface at the desired operating wavelength. Similarly, the constructed system is capable of achieving highly accurate polarization measurements at other operating wavelengths throughout the visible light range, by simply replacing the narrow-band filter at the front end of the system, without necessitating the re-fabrication of the metasurface device. We perform polarization measurements using the same metasurface at other visible wavelengths, such as 450 nm and 650 nm. Through recalibration and reconstruction, the experimental results still demonstrate a high level of measurement accuracy. Details are provided in Supplementary Material S3.

### Single-shot full-Stokes polarization imaging

**Fig. 5:**
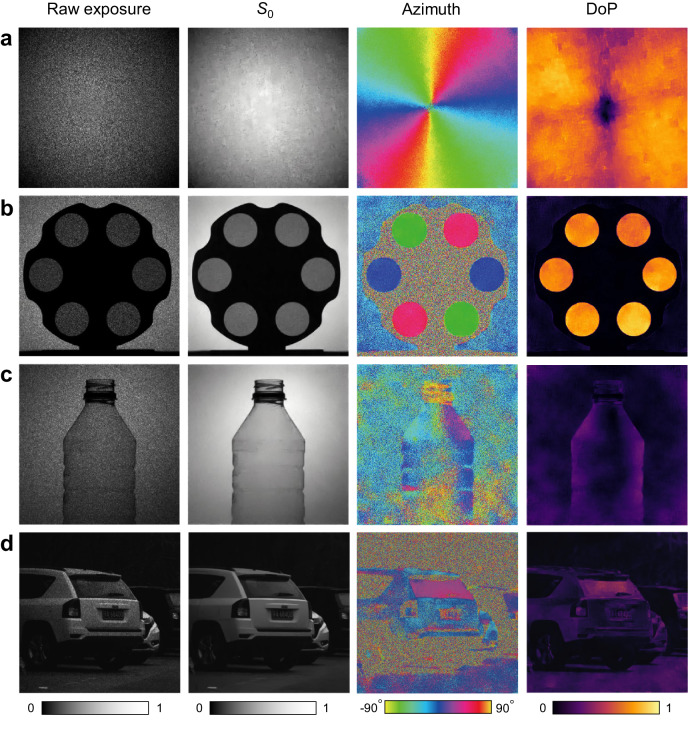
Full-Stokes polarization imaging. Indoor (**a**–**c**) and outdoor (**d**) photography with the proposed metasurface camera. In each case, the raw exposure (corresponding to the polarization-encoded image, or called compressed image), *S*_0_ (corresponding to the monochrome intensity image), the azimuth of the polarization ellipse and the degree of polarization are shown.

To validate the single-shot polarization imaging functionality, we show several photographs captured by the metasurface polarization camera equipped with a 10 nm bandpass filter centered at 550 nm (Fig. 5). For each, images of the raw acquisition on the sensor, *S*_0_, the azimuth angle and the DoP are shown. The scene in Fig. 5a is a *q*-plate. In this experiment, an incoherent light beam is transmitted through a linear polarizer and the *q*-plate is used to generate a polarized vector beam. From the intensity image (*S*_0_), the polarization information of the incident beam could not be observed. The azimuth image, however, displays smooth and continuous azimuth angle evolution around the center point, and the DoP image further reveals a singularity at the center. Figure 5b, c show the indoor photographs taken with ambient unpolarized light. The scene in Fig. 5b describes a filter wheel with six sheets of film polarizer whose axes are arranged radially outward. The intensity image *S*_0_ cannot recognize the difference between the six polarizers, but the azimuth image accurately reveals their angular orientations. Moreover, the DoP image indicates that unpolarized light passing through the film polarizers also becomes purely polarized. In Fig. 5c, we took the photograph of a plastic bottle. Although its 3D morphology is not discernible from the intensity image *S*_0_, the azimuth and DoP images evidence its cylindrical shape due to the distinct polarization responses of the plastic surface texture to the incident light. Figure 5d shows an outdoor photograph taken in the daytime at a parking lot. For all three images, a sport utility vehicle can be clearly seen. Compared with the intensity image *S*_0_, the afterbody and rear windshield of the vehicle are highly prominent relative to the background in the azimuth and DoP images, which originates from the fact that the metallic body and windshield of the vehicle tend to yield strong polarization response. Moreover, even at the pixel level, scenes such as the numbers on a car plate remain highly distinct and discernible. Details can be found in Supplementary Fig. 13.

**Fig. 6:**
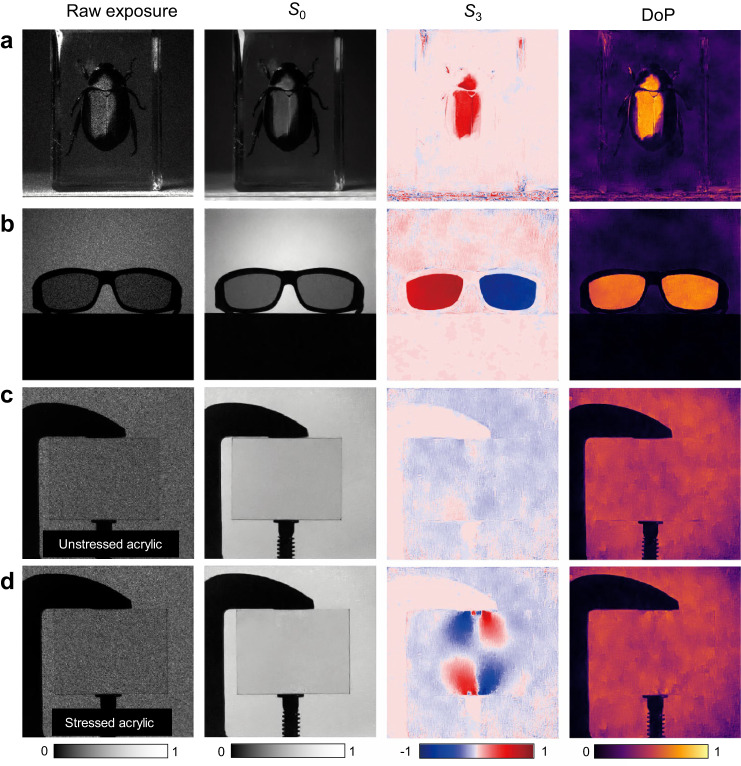
Polarization imaging of *S*_3_. In each example, raw sensor acquisition, *S*_0_*, S*_3_ and DoP are shown. **a** Biological specimen. **b** 3D glasses consist of opposite circular polarization filters. **c** A rectangle acrylic piece is not stressed by the clamp-squeezing and displays no stress-induced birefringence in the *S*_3_ image. **d** The rectangle acrylic piece is stressed by clamp-squeezing and displays stress-induced birefringence in the *S*_3_ image.

In these experiments, although the *S*_3_ components (circular polarization signatures) are contained in DoPs, they cannot be directly observed due to the chiral insensitivity of the target objects. In fact, most existing commercial polarization cameras are unable to measure *S*_3_. However, our proposed single-shot metasurface camera is capable of measuring *S*_3_ and achieves full-Stokes polarization imaging, as shown in Fig. 6. Figure 6a shows a large biological specimen, *anomald corpulenta motschulsky*, which is a beetle that naturally exhibits strong circular dichroism for visible light. The circular dichroism can be clearly observed in the *S*_3_ image, as the exoskeleton of beetle strongly reflects one of the chiral components while absorbing the other. Since *S*_3_ is a positive value, we can infer that the primary component of the reflected light is right-handed circular polarization state. Figure 6b depicts a pair of 3D glasses whose frames containing opposite circular polarizers. This is not visible in the *S*_0_, but can be clearly seen in the *S*_3_ image where each lens has a ±1 value. In Fig. 6c, d, a rectangular piece of acrylic is illuminated from behind using uniform linearly polarized light from a liquid crystal display. In the loose state of the clamp, the reconstructed *S*_3_ image displays little chiral component (Fig. 6c). Upon squeezing (Fig. 6d), a wealth of information is obtained in the *S*_3_ image stemming from stress-induced birefringence, which is invisible to the human eye or a traditional camera. Visualization of stress field is a unique advantage of full-Stokes polarization imaging, which can find widespread applications in machine vision, stress measurement and defect detection.

## Discussion

Previously reported imaging polarimetry always rely on rigorous design and precise fabrication of nanostructures to obtain strong dichroism^26–38^. This comes from the common belief that polarimetry accuracy significantly depends on the diattenuation of the polarization components. However, in the proposed nanophotonic polarization camera, the imaging process relies on a pre-calibrated compressive sensing matrix derived from an array of disordered metasurfaces, along with a mask-aware reconstruction algorithm. As a result, there is no need for a strong dichroism. Our approach not only significantly improves the accuracy of polarization measurement, but also greatly relaxes the design and fabrication requirements on meta-optics. The proposed reconstruction algorithm could automatically extract statistical characteristics from the input dataset and performs effectively on pixel-level natural scenes. Since trained in a mask-aware manner, the reconstruction algorithm can also be flexibly applied to other types of polarization imaging systems with a pre-calibrated three-dimensional transmission matrix **M**, enabling higher applicability and scalability for practical applications. The reconstruction algorithm with the proposed neural network is capable of real-time inferencing an image using a commercial graphical processing unit, which enables dynamic scene recording. Further experimental results on polarization camera, including the reconstruction algorithm, operation at different wavelengths within the visible spectrum, and real-time polarization imaging of a dynamic scene, are given in Supplemental Material S4 and Supplementary Movie 1.

The advantage presented by this statistical approach hinges on priors learned from a dataset of acquired scenes. If the polarized scenes being sensed contain sufficient sparse priors, the combination of metasurfaces and algorithms presented in this work can yield a resolution advantage over other polarimetry schemes (see Fig. 3). By retraining on datasets from other domains, the sparsity prior memorized in neural network will be updated and the reconstruction algorithm can be easily generalized to other special scenes, such as remote sensing and microscopy, where the physics at-play may yield different priors. However, as a statistical method, the proposed approach may yield unreliable results when reconstructing data from certain domains where these priors have not been learned or where such sparsity does not exist in the first place.

In conclusion, we demonstrate a single-shot full-Stokes polarization camera incorporating a disordered metasurface array exhibiting weak dichroism and a mask-aware, deep compressed sensing algorithm. It exploits the unprecedented ability of disordered pixelated metasurface design to efficiently manipulate the polarization of incident optical waves. The flexible and compact system framework, accurate polarimetry performance and fast reconstruction algorithm show great potential for this nanophotonic polarization camera in various applications including but not limited to microscopy, augmented reality, machine vision and remote sensing.

## Methods

### Nanofabrication of metasurface array

A layer of positive electron-beam resist is spin-coated onto the fused silica substrate, followed by baking on a hot plate. The spin-coating speed is adjusted to yield a resist thickness of 600 nm. To suppress charging during electron beam lithography, a 15-nm thick aluminum (Al) layer is thermally evaporated onto the resist layer. The pattern of metasurface array is defined in resist using an electron-beam lithography system (beam current of 2 nA and accelerating voltage of 100 kV), followed by Al layer removal and resist development. The patterned resist is then coated with a layer of TiO_2_ using atomic-layer deposition (ALD) system. This process is done at a low temperature of 90 °C to prevent deformation of the resist pattern. After the ALD step, the overcoated TiO_2_ film is removed using an inductively coupled plasma reactive ion etching system. The etching is stopped until the overcoated TiO_2_ layer is completely removed and the resist is exposed. Finally, the remaining resist is removed by soaking in n-methyl-2-pyrrolidone, yielding the array of TiO_2_ nanopillars with smooth and straight sidewall profiles.

### Polarimetric measurement procedure

The optical setup of the polarimetry measurements is shown in Supplementary Fig. 7a. In our experiment, a 16 × 16 meta-pixel array is utilized as a single-point polarimeter, with spatial dimensions of ~110 um × 110 um, as shown in Supplementary Fig. 7b, c. A uniform LED light is emitted from an integrating sphere and enters the system through a linear polarizer, spectral filter (10 nm bandpass filter) and retarder. By rotating the angle of the linear polarizer and the fast axis of the retarder, arbitrary SoP can be generated. Subsequently, the incident light passes through the disordered metasurface array and is ultimately captured by the sensor using a relay lens. Simultaneously, the SoPs of incident beam are also monitored with a commercially available rotating-wave-plate polarimeter as reference.

### Supplementary information

Supplementary Information

Description of Additional Supplementary Files

Supplementary Movie S1

## Data Availability

All the processed data in this study are provided within the paper and its supplementary information. The data that support the findings of this study are available from the corresponding author upon request.

## Code availability

The code used for analyses is available from the corresponding author upon request.
