# Supplementary material for: Disordered metasurface enabled single-shot full-Stokes polarization imaging leveraging weak dichroism
Source: Nat Commun. 2023 Nov 7;14:7180. doi: 10.1038/s41467-023-42944-6 (PMC10630513; doi:10.1038/s41467-023-42944-6)
Supplement: Supplementary file 1 — Supplementary Information [file 41467_2023_42944_MOESM1_ESM.pdf]

Supplementary Information for "Disordered metasurface enabled single-shot full-Stokes polarization imaging leveraging weak dichroism"

Qingbin Fan[1,2,3], Weizhu Xu[1,2], Xuemei Hu[1,2], Wenqi Zhu[4,5], Tao Yue[1,2], Feng Yan[1,2], Peicheng Lin[1,3], Lu Chen[4,5], Junyeob Song[4], Henri J. Lezec[4], Amit Agrawal[4], Yanqing Lu[1,2,3] and Ting Xu[1,2,3]

1. National Laboratory of Solid-State Microstructures and Collaborative Innovation Center of Advanced Microstructures, Nanjing University, Nanjing 210093, China
2. School of Electronic Sciences and Engineering, Nanjing University, Nanjing 210093, China
3. College of Engineering and Applied Sciences and Jiangsu Key Laboratory of Artificial Functional Materials, Nanjing University, Nanjing 210093, China
4. Physical Measurement Laboratory, National Institute of Standards and Technology, Gaithersburg, Maryland 20899, United States
5. Maryland NanoCenter, University of Maryland, College Park, Maryland 20899, United States

CONTENTS

S1. Details of the disordered metasurface array 1

S2. Sampling efficiency 4

S3. Full-Stokes polarimetric measurements 5

A. Mathematical exposition 5

B. Polarimetry measurements at other wavelengths 5

S4. Full-Stokes polarization imaging 8

A. Mathematical exposition 8

B. Deep mask-aware compressed sensing network 8

C. Experimental Setup 11

D. Polarization imaging at other wavelengths 12

E. Description for supplemental movie S1 12

Supplementary References 26

S1. Details of the disordered metasurface array

The pixelated disordered metasurface consists of a single layer array of subwavelength dielectric nanopillars. An unpolarized optical wave which can be considered as a combination of any pair of orthogonal SoPs is incident on the metasurface. In addition to polarization and phase, the amplitudes of a pair of orthogonal SoPs through each meta-pixel can be controlled independently via judicious pixel design [1]. In this work, a meta-pixel array is composed of two distinct anisotropic nanopillars. The interleaved arrangement of these two nanopillars forms a miniature interference system, which plays a critical role in achieving polarization-dependent amplitude modulation. Specifically, each nanopillar can be considered as an individual dielectric waveguide that supports different optical modes along the rectangular nanopillars' two symmetry axes. As a result, the two nanopillars impose polarization dependent complex amplitudes $U_a^{\pm}(x,y)$ and $U_b^{\pm}(x,y)$ to the transmitted optical waves. The total complex amplitude of the miniature interference system is given by $U^{\pm}(x,y) = U_a^{\pm}(x,y) + U_b^{\pm}(x,y)$, where $+$ and $-$ denote a pair of orthogonal states of polarization. By altering the phase difference $\varphi^{\pm}(x,y)$ of the complex amplitudes between the two nanopillars, any polarization-dependent transmitted-light amplitude $|U^{\pm}(x,y)|$ can be achieved.

There are two kinds of wavefront modulation mechanisms for polarization-dependent dielectric metasurfaces including propagation phase and geometric (or Pancharatnam-Berry, PB) phase [2]. Propagation phase designs are dependent on the shape of the nanopillars, which allow for the metasurface imparting two independent spatial phase profiles on a pair of orthogonal, linear polarizations. Geometric phase designs are only determined by angular orientation (θ) of the nanopillar, which allow for the imposition of

two equal but opposite phase profiles ($\varphi^{\pm} = \pm 2\theta$) on a pair of orthogonal, circular polarizations.

The designed metasurface device consists of rectangular titanium dioxide (TiO_2) nanopillars with different dimensions and orientations, resting on a fused-silica substrate. The nanopillars are designed to have identical heights of h=600 nm and are arranged in a square lattice. Here, we provide a simple example to illustrate the design process and optical properties of the constructed meta-pixel. As shown in Supplementary Fig. 1a, the meta-pixel design starts from an anisotropic nanopillar. The simulation results exhibit a linear polarization-dependent optical response. Using the geometric phase alone (Supplementary Fig. 1b), the transmitted-light intensities of a pair of orthogonal circular SoPs decrease simultaneously since the almost identical interference effect occurs on both circular polarizations. Moreover, the circular polarization-dependent optical response is quite weak and difficult to control. Here, a unification of the propagation phase design and geometric phase design is employed to achieve the polarization-dependent interference behavior for any pair of orthogonal SoPs (Supplementary Fig. 1c and Supplementary Fig. 2), including linear and circular polarizations. Supplementary Fig. 3 shows optical properties of the chosen nanopillars and the constructed meta-pixel over the entire visible spectral range from 400 nm to 700 nm. The simulation results demonstrate that the proposed dielectric meta-pixel has capability of providing a desired effect of polarization-dependent transmission for any pair of orthogonal SoPs. The output signals from the different meta-pixels are collected by a detector and then processed to reconstruct the incident SoP.

In our design, the first task is to perform a series of parameter sweeps and establish a library that determines the relation between birefringent phase shifts (δ_x, δ_y) and lateral dimensions (D_x, D_y) of a rectangular nanopillar. The height of the nanopillars is fixed at 600 nm, while the lateral dimensions, period, and central wavelength are considered as adjustable design parameters. The period varies between 300 nm to 450 nm, and the central wavelength ranges from 400 nm to 700 nm, forming a large parameter space. Supplementary Fig. 4 gives an example which shows simulated transmitted-light intensity and phase shifts of transmission coefficients for an infinite periodic array of rectangular nanopillars at a wavelength of 550 nm. The nanopillar size is varied from 80 nm to 330 nm in a square lattice of 400 nm period. The simulations are implemented by employing a finite-difference-time-domain (FDTD) method with linearly polarized light illumination from the SiO_2 substrate side. To increase the reconstruction performance across the whole visible spectrum, 512 nanopillars are chosen as the basic building blocks for the metasurface array. Each meta-pixel contains a periodic array of two kinds of anisotropic nanopillars with varying orientations and sizes. In total, there are 256 distinct meta-pixels forming the entire metasurface device.

In the main text, we employed diattenuation as a metric to evaluate the designed metapixels. In general, diattenuation refers to the polarization-dependent transmittance of a material, where the amount of transmitted light varies depending on the incident polarization state. For the designed metapixel, the Stokes-like vector $\boldsymbol{M}_{i,j}$ contains parameters $(m_{i,j}^0, m_{i,j}^1, m_{i,j}^2, m_{i,j}^3)$. The first element of $\boldsymbol{M}_{i,j}$ gives average power of a pair of orthogonal SoPs and the last three elements describe the polarization-dependence features. The diattenuation of a metapixel is determined by comparing the magnitudes of

the last three elements with respect to the first. Specifically, the diattenuation of the designed metapixel can be obtained using the following formula:

$$D = \frac{1}{m_{i,j}^{0}} \sqrt{m_{i,j}^{1} + m_{i,j}^{2} + m_{i,j}^{3}} \tag{S1}$$

In our method, the imaging device we used exhibits low diattenuation, a distinctive characteristic that sets it apart from conventional imaging techniques. On the other hand, the disordered metasurfaces provide ‘randomness’ in spatial and polarization dimensions (Supplementary Fig. 5), enabling them to serve as effective sensing matrices for high-precision signal reconstruction.

S2. Sampling efficiency

In this section, we provide a detailed calculation process regarding the sampling efficiency for the comparison of different arrangements [3-5]. According to the principles described in the main text, the smaller $\mu(\boldsymbol{\Lambda}, \boldsymbol{\Psi})$, the fewer sampling points are required, thereby indicating a higher sampling efficiency of the sensing system. The sparse representation $\boldsymbol{\Psi}$ is signal dependent, and can be learned from the dataset. In the calculation of sampling efficiency, $\boldsymbol{\Psi}$ is derived from a full-Stokes polarization image dataset [6] by employing the PCA method. The pipeline is shown in Supplementary Fig. 6. Specifically, we divide the polarization dataset into 21175 patches of $12 \times 12 \times 4$ pixels (horizontal × vertical × polarization). As for $\boldsymbol{\Lambda}$, we selected 10000 sampling patches of the measurement matrix $\boldsymbol{M}$ with a size of 12×12 pixel to evaluate the sampling efficiency of three DoFP arrangements. The sensing matrix $\boldsymbol{\Lambda}$ is uniformly defined as $\boldsymbol{\Lambda} = \mathrm{diag}(\boldsymbol{M}_{1,1}^{T}, \boldsymbol{M}_{1,2}^{T}, \ldots, \boldsymbol{M}_{12,12}^{T})$. The size of the sparse representation matrix $\boldsymbol{\Psi}$ and the sampling matrix $\boldsymbol{\Lambda}$ are 576×576 and 144×576, respectively. We tested the sampling efficiency of different arrangements and

depicted the results of the sampling efficiency metric $\mu(\boldsymbol{\Lambda}, \boldsymbol{\Psi})$ in Fig. 3d. The sampling efficiency of our disordered metasurface array is comparable to that of conventional ordered sampling schemes, implying the potential of the proposed disordered sampling scheme for high performance polarization imaging.

S3. Full-Stokes polarimetric measurements

A. Mathematical exposition

As described in the main text, the transmitted light intensities $\boldsymbol{I}$ captured by the image sensor can be modeled by:

$$\boldsymbol{I}_v = \boldsymbol{\Lambda}_{dot}\boldsymbol{S} + \boldsymbol{N}, \tag{S2}$$

where $\boldsymbol{I}_v$ denotes the vectorized measurement, $\boldsymbol{\Lambda}_{dot} = [\boldsymbol{M_1}, \boldsymbol{M_2}, \dots, \boldsymbol{M_m}]^T$ denotes the polarization sensing matrix of a single micro-polarimeter, $\boldsymbol{S}$ denotes the full-Stokes parameters of the uniform incident light, $\boldsymbol{N}$ represents the noise and m is the meta-pixel number of measurements utilized for the single polarimeter. When m is larger than four, Eq. S2 becomes overdetermined and can be easily solved by the damped least square method [7]:

$$\boldsymbol{S} = (\boldsymbol{\Lambda}_{dot}^T\boldsymbol{\Lambda}_{dot} + \eta\boldsymbol{E})^{-1}\boldsymbol{\Lambda}_{dot}^T\boldsymbol{I}_v\ , \tag{S3}$$

where $\boldsymbol{E}$ and η denote the identity matrix and the damped factor, respectively.

B. Polarimetry measurements at other wavelengths

We also perform polarization measurements using the same metasurface at other visible wavelengths, such as 450 nm and 650 nm. Supplementary Fig. 7 shows the optical setup of the polarization measurements. Supplementary Figs. (8-9) show the experimental results. By summarizing the reconstructed Stokes parameters of 25 arbitrarily selected

SoPs at a wavelength of 650 nm, the average measurement errors of S_1, S_2, and S_3 are $\pm 0.46\%$, $\pm 0.44\%$, and $\pm 0.43\%$, respectively. At a wavelength of 450 nm, the average measurement errors of S_1, S_2, and S_3 are $\pm 1.10\%$, $\pm 0.71\%$, and $\pm 0.80\%$, respectively. Obviously, the experimental results still demonstrate a high level of measurement accuracy. Compared with other metasurface-based polarimetric detection techniques reported in the literature, our method exhibits the higher performance in measurement accuracy (summarized in Table S1).

Table S1 | Full-Stokes polarimetric measurement techniques

Items / Refs	Structure design	Normalized Operation Bandwidth $((\lambda_{max} - \lambda_{min})/\lambda_0)$ ①	Error of Stokes parameters ②
Ref. 8	SPP excitation by X-shaped aperture Array	33.3% (750-1050 nm)	≈10% (S_1), ≈17% (S_2), ≈12% (S_3)
Ref. 9	Metasurface in-line polarimeter	≈4.2% (1500–1565 nm)	≈6% (S_1), ≈6% (S_2), ≈5% (S_3)
Ref. 10	Spin–orbit interaction of light with scatterers	≈6.5% (1.5–1.6 μm)	≈7% (S_1), ≈16% (S_2), ≈11% (S_3)
Ref. 3	Dielectric metasurface	Narrow bandwidth (≈ 850 nm)	7.5–13% (S_1-S_3)
Ref. 11	Vertically stacked metal-dielectric hybrid metasurfaces	≈10%(1.4–1.55 μm)	1.9% (S_1), 2.7% (S_2), 7.2% (S_3)
Ref. 12	Liquid crystal	NA	780 nm: 12.4% (S_1), 14.1% (S_2), 1.7% (S_3)
Ref. 13	Vertically stacked plasmonic metasurfaces	≈7.6%(3.8–4.1 μm)	4 μm: 3.5% (S_1), 2.5% (S_2), 10.4% (S_3)
Ref. 14	Plasmonic metagrating	35.3%(700–1000 nm)	≈10% (S_1-S_3)
This paper	Disorder metasurface with weak dichroism	54.5% (400-700 nm)	650 nm: 0.46% (S_1), 0.44% (S_2), 0.43% (S_3)<br>550 nm: 0.50% (S_1), 0.58% (S_2), 0.48% (S_3)<br>450 nm: 1.10% (S_1), 0.71% (S_2), 0.80% (S_3)

①λ_0 denotes center wavelength. ② Errors for the Stokes parameters are the arithmetic mean extracted from the data presented in each article.

The polar plot representation in Fig.4 and Supplementary Figs. (8-9) provides a more intuitive way to describe the properties of a polarization state, such as the azimuth of the polarization ellipse and radiant intensity in specific polarization directions. To create a polar plot representation of a polarization ellipse, we can convert the equation of the ellipse expressed in Cartesian coordinates (x, y) to polar coordinates (ρ , θ) by using the

relationship: $x = \rho\ cos\theta$ and $y = \rho\ sin\theta$. In our work, the polar coordinate system is set up as (ρ^2, θ), where ρ^2 intuitively represents the intensity of polarized light waves when passing through a linear polarizer with an azimuth angle of θ.

To further examine the performance of the metasurface polarimeter with respect to a benchmark polarization element, we measured the Stokes vector of a beam passing through a Glan-Thompson polarizer at the wavelength of 550 nm. By analyzing the reconstructed Stokes parameters of 15 arbitrarily selected SoPs, the average measurement errors of S_1, S_2, and S_3 are ±0.53 %, ±0.68 %, and ±0.47 %, respectively. The reconstruction error for DoP is ±0.41 %. The experimental results also demonstrate that our method can achieve a high measurement accuracy, which is comparable to the results obtained using a wire-grid polarizer in our previous experiments. This conclusion is fully justified based on the following reasons: In the presence of photon noise, the polarimetric error is inversely proportional to the diattenuation of the polarization optics being used. A high-quality Glan-Thompson polarizer and a wire grid polarizer can provide comparable performance in a Stokes vector measurement since these two both have a diattenuation exceeding 99.9%. Therefore, the results obtained using these two polarizers are nearly identical.

S4. Full-Stokes polarization imaging

In this section, the image formation model and the corresponding deep mask-aware compressed sensing algorithm of the full-Stokes polarization imaging will be introduced.

A. Mathematical exposition

As described in the main text, the captured image can be written as:

$$\boldsymbol{I}_v = \boldsymbol{\Lambda}\boldsymbol{S}_v + \boldsymbol{N}, \tag{S4}$$

where $\boldsymbol{I}_v$ represents the vectorized form of $\boldsymbol{I}$, $\boldsymbol{\Lambda} = \mathrm{diag}(\boldsymbol{M}_{\boldsymbol{1},\boldsymbol{1}}^{\boldsymbol{T}}, \boldsymbol{M}_{\boldsymbol{1},\boldsymbol{2}}^{\boldsymbol{T}}, \dots, \boldsymbol{M}_{\boldsymbol{m},\boldsymbol{n}}^{\boldsymbol{T}})$ is the polarization transmission matrix in diagonal form, $\boldsymbol{S}_v = \left[\boldsymbol{S}_{1,1}^{\boldsymbol{T}}, \boldsymbol{S}_{1,2}^{\boldsymbol{T}}, \dots, \boldsymbol{S}_{\boldsymbol{m},\boldsymbol{n}}^{\boldsymbol{T}}\right]^T$ denotes the target full-Stokes polarization images in vectorized form, $\boldsymbol{N}$ denotes the noise. We form the polarization image as a three-dimensional (space *x*-*y* and polarization *p*) full-Stokes polarization sensing problem with compressed two-dimensional (space *x*-*y*) measurements. The reconstruction process could be formulated as:

$$\boldsymbol{S}_v = \mathrm{argmin}\|\boldsymbol{\Lambda}\boldsymbol{S}_v - \boldsymbol{I}_v\|_2^2 + \eta\Gamma(\boldsymbol{S}_v) , \tag{S5}$$

where term Γ is a regularizer determined by the prior knowledge of the input scene $\boldsymbol{S}_v$ (e.g., sparsity), and term η is the weight for the prior knowledge. In this paper, to fully exploit the statistical prior of natural images and incorporate the information from the calibrated compressive polarization sensing matrix, we propose a deep mask-aware compressed sensing network for high fidelity full-Stokes reconstruction.

B. Deep mask-aware compressed sensing network

As described in section A, the image reconstruction is an inverse problem of Eq. S4. A straightforward approach involves generating the training data pairs (the polarization-encoded image $\boldsymbol{I}$ and full-Stokes images $\boldsymbol{S}$) from the full-Stokes polarization dataset and

the 3D transmission matrix $\boldsymbol{M}$ obtained by calibration, and then training the deep neural network as a mapping from $\boldsymbol{I}$ to $\boldsymbol{S}$. However, without inputting the information of the transmission matrix $\boldsymbol{M}$, the trained model will suffer from overfitting risk and can only infer on imaging systems with the same transmission matrix. To overcome these problems, we generate random 3D transmission matrix for each full-Stokes polarization image to synthesize the polarization-encoded image $\boldsymbol{I}$ based on Eq. S4 and introduce the corresponding random transmission matrix as another input into the network as shown in Supplementary Fig. 10. Each pair of training data is coded with a unique random transmission matrix and the network is trained in a mask-aware way. Thus, the proposed deep mask-aware compressive network will tend to learn the solution of Eq. S4 instead of a fixed mapping between two fixed training datasets generated with a fixed transmission matrix, enabling higher applicability and scalability for practical applications.

Network architecture: The proposed neural network adopts the fully convolutional neural network structure (CNN), which consists of 10 residual blocks. The captured image $\boldsymbol{I}$ and random transmission matrix $\boldsymbol{M}$ are concatenated into a size of 128×128×5 cube as the input of the neural network. The output is the full-Stokes polarization images $[S_0, S_1, S_2, S_3]$. The specific structure of the network is shown in Table S2.

A compound loss function, consisting of the mean square error (MSE) and L_1 norm, is used to improve the quality of the results:

$$L_{\text{total}} = \|\boldsymbol{G} - \mathrm{F}(\boldsymbol{I}, \boldsymbol{M})\|_2^2 + \lambda \|\boldsymbol{G} - \mathrm{F}(\boldsymbol{I}, \boldsymbol{M})\|_1 \, , \tag{S6}$$

where $\boldsymbol{G}$ denotes the ground truth image and F denotes the corresponding operator of the neural network. The weight of L_1 norm λ is empirically set to 1.

Table S2. Architecture of the deep compressed sensing -based neural networks

Name	Structure	Kernel size	Stride	Parameters
Input layer	Conv	5×64×5×5	1	8K
Body	Res-block × 10	64×64×5×5	1	2.048M
Output layer	Conv	64×4×5×5	1	6.4K
Res-block	Conv	64×64×5×5	1	102.4K
	ReLU	-	-	0
	Conv	64×64×5×5	1	102.4K

Dataset: Since there is currently a lack of full-Stokes polarization image datasets with enough scales for training deep neural networks, we propose to construct the training dataset by combining channels of similar multi-channel image datasets, i.e., the hyperspectral dataset. In the experiment, we randomly sample 26,016 hyperspectral patches with 31 channels from five common hyperspectral datasets [15-20]. The spatial size of these patches is set as 128×128. Then seven channels of these patches are selected randomly, denoted by $I_{1\sim7}$. The four-channel full-Stokes image $\boldsymbol{S} = [S_0, S_1, S_2, S_3]^T$ can be computed by $S_0 = I_1, S_1 = I_2 - I_3, S_2 = I_4 - I_5, S_3 = I_6 - I_7$. According to the generating method, although few non-existing polarization vectors (e.g., some points may not be located within the Poincaré sphere) may be involved, all the naturally existing polarization states could be fully covered and the neural network with training data generated from this dataset could be readily effective for real-world proposed polarization imaging. To further increase the robustness of the algorithm, we added 5% Gaussian white noise to both the transmission matrix $\boldsymbol{M}$ and polarization-encoded image $\boldsymbol{I}$ during the training process as shown in Supplementary Fig. S10. With these data enhancement strategies, the network

learns to reconstruct high-fidelity full-Stokes polarization information despite the noise deteriorations during the imaging process.

Training: The network is implemented upon the Pytorch platform. We train the network by the Adam optimizer with $\beta_1 = 0.9$ and $\beta_2 = 0.999$. The total number of training epoch is set to be 100. The learning rate is initially set to 10^{-4} within the first 50 epochs and decayed to 10^{-5} in the last 50 epochs. The batch size is set to 32. The whole training procedure takes approx. 12 h on a commercial graphics processing unit (Nvidia Geforce RTX 3090).

Testing After training, the full-Stokes polarization images can be reconstructed from the captured images with their corresponding 3D transmission matrix. The trained neural network contains 2.06 M parameters and requires 330.3 G Flops for inferencing a 400×400 image. In terms of reconstruction speed, the network is capable of inferring full-Stokes images from raw data at a frame rate of 26 frames per second (FPS) on a commercial graphics processing unit (Nvidia GeForce RTX 3090).

C. Experimental Setup

Supplementary Fig. 11 shows the optical setup of the proposed polarization imaging system. The prototype system is composed of a commercial primary lens (focal length: 25mm), a filter with a bandwidth of 10nm at target spectrum, the designed metasurface array and a relay imaging system consisting of a 10× objective lens (Olympus RMX) and an imaging sensor (Grasshopper3 GS3-U3-123S6C). It’s worth noting that the system operates with a single shot, eliminating the need for mechanical movement. Although the system used for calibration (Supplementary Fig. 7) differs from the one used for polarization imaging (Supplementary Fig. 11), it has no impact on our imaging performance. This is because the

matrix elements recovered from calibration are only related to the optical properties of the device itself, and are independent of the light sources and optical elements in the system.

D. Polarization imaging at other wavelengths

In our approach, the proposed polarization camera can retrieve the SoP of incident light at other wavelengths within the visible light range, spanning from 400 nm to 700 nm. For different operating wavelengths, only the color filter needs to be replaced. We select several representative wavelengths, namely 450 nm, 500 nm, 550 nm, 600 nm, and 650 nm, for experimental verification. As depicted in Supplementary Fig. 12, the proposed camera consistently maintains high image quality at different wavelengths.

E. Description for supplemental movie S1

The scene in movie describes a filter wheel fitted with six sheets of film polarizer whose axes are arranged radially outward. The filter wheel is driven by an electric motor. The raw unprocessed exposure (top left), S_0 (top right), DoP (bottom left), and the azimuth of the polarization ellipse (bottom right) are shown. Illumination is provided by a LED with a 10 nm bandpass filter centered at 550 nm.

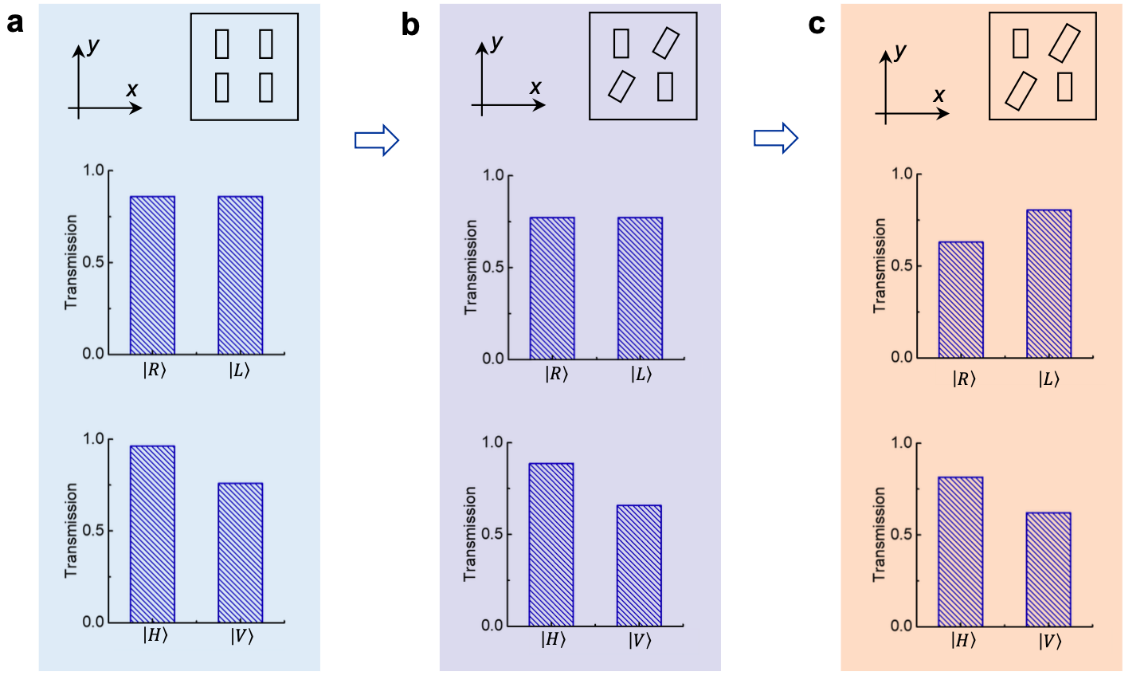

Supplementary Fig. 1 | Design of polarization-dependent meta-pixel. (**a**) Polarization response of anisotropic nanopillars in an infinitely periodic array. Nanopillars have: (D_x=85 nm, D_y =230 nm, $\Delta\theta$=0°). Here, $\Delta\theta$ represents relative rotation angle between two nanopillars. (**b**) Using identical nanopillars with a fixed $\Delta\theta$−that is geometric phase design. Nanopillars have: (D_x=85 nm, D_y =230 nm, $\Delta\theta$=30°). (**c**) By varying both dimensions and rotation angle−that is by combining the propagation and geometric phase design. Nanopillars have: #1(D_x=85 nm, D_y =230 nm), #2(D_x=105 nm, D_y =245 nm), and $\Delta\theta$=30°.

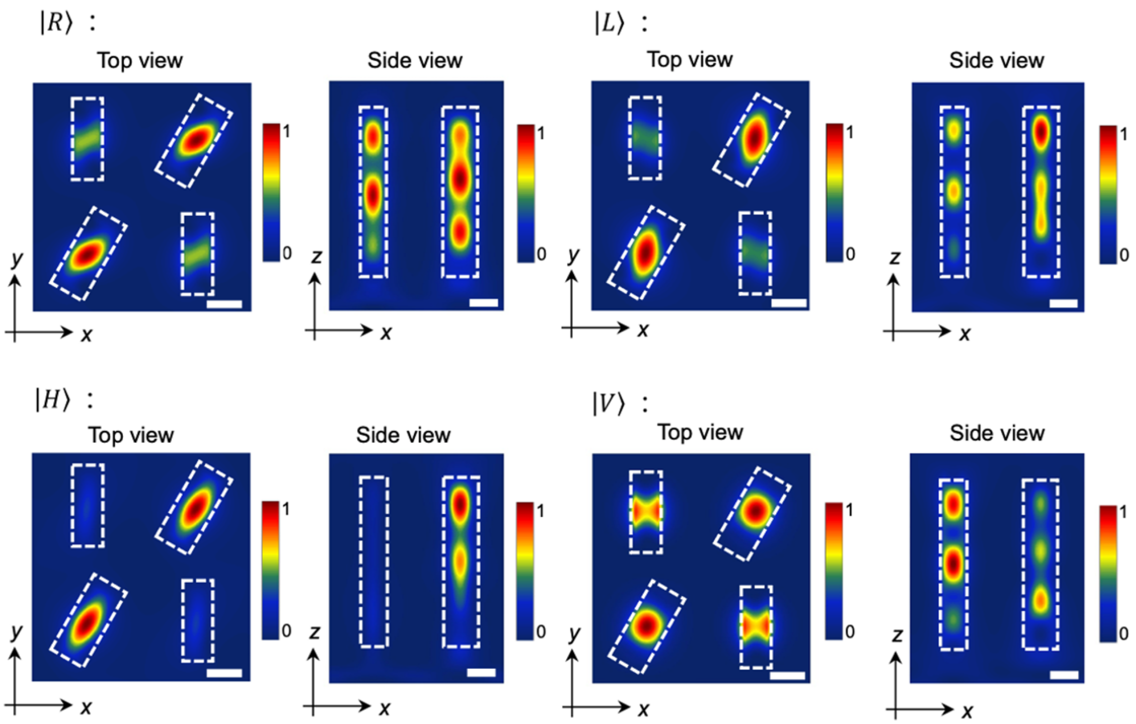

Supplementary Fig. 2 | Simulated energy density of polarization-dependent meta-pixel. Top views and side views of the normalized energy density in an infinitely periodic array for the designed meta-pixel in Supplementary Fig. 1c. The boundaries of the nanopillars are depicted by dashed white lines. Scale bars represent 100 nm in all figures.

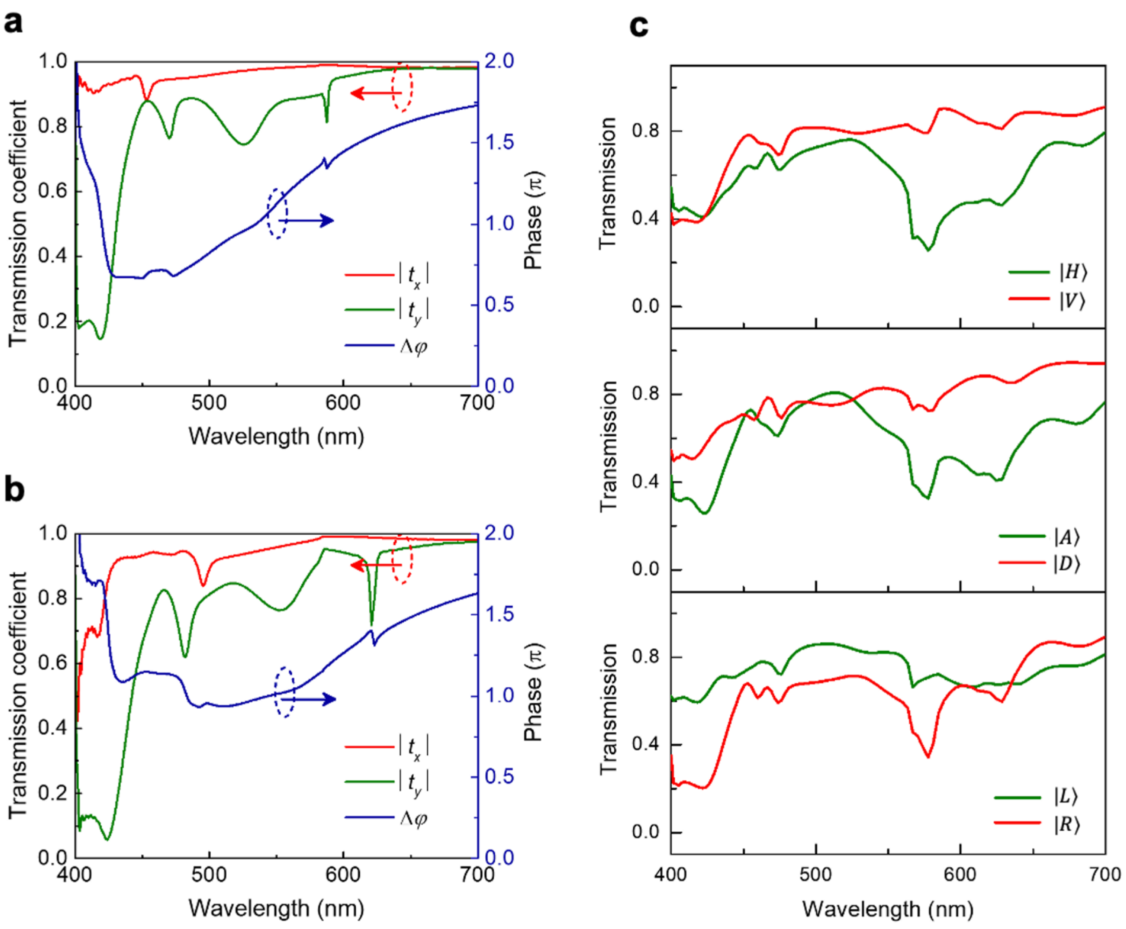

Supplementary Fig. 3 | Optical properties of the designed nanostructures. (**a** and **b**) Calculated transmission coefficients (red $|t_x|$; green $|t_y|$) for horizontally and vertically polarized light and their phase difference (blue, $\Delta\varphi$). In **a**, nanopillars have: (D_x=85 nm, D_y =230 nm). In **b**, nanopillars have: (D_x=105 nm, D_y =245 nm). (**c**) Polarization-dependent transmission of the designed meta-pixel in Supplementary Fig. 1c.

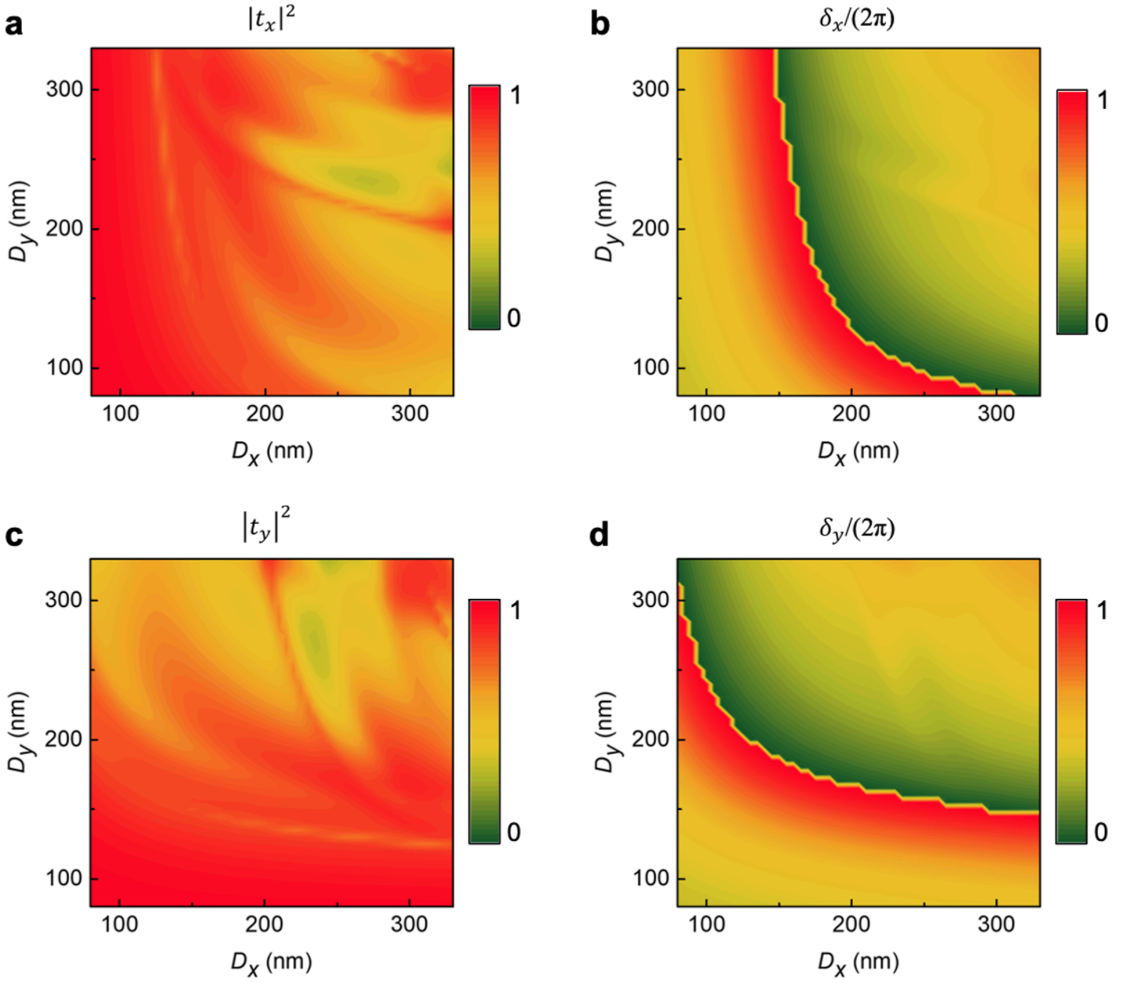

Supplementary Fig. 4 | Simulation results for parameter space (axis length D_x and D_y) of rectangular TiO_2 nanopillars. Simulated transmitted-light intensity (**a**. $|t_x|^2$, **c**. $|t_y|^2$) and phase shifts (**b**. δ_x, **d**. δ_y) of transmission coefficients for an infinitely periodic array of the rectangular nanopillars. The nanopillars are varied from 80 nm to 330 nm in a square lattice with a 400 nm period. In these simulations, the nanopillars are 600 nm tall and the wavelength is 550 nm.

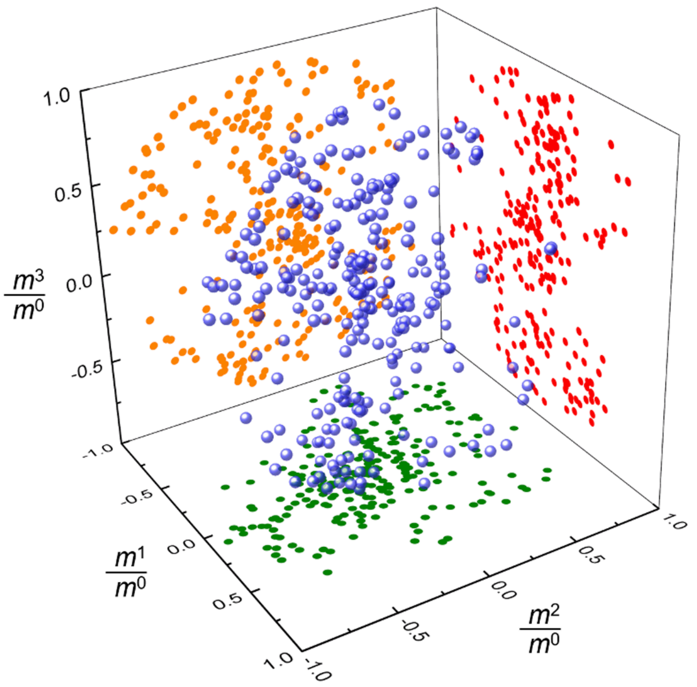

Supplementary Fig. 5 | The distribution of transmission vector $M_{i,j}$ of 256 different meta-pixels. The bule points denote the normalized distribution of different meta-pixel. The red, green and orange points are the projection of the bule points at different planes.

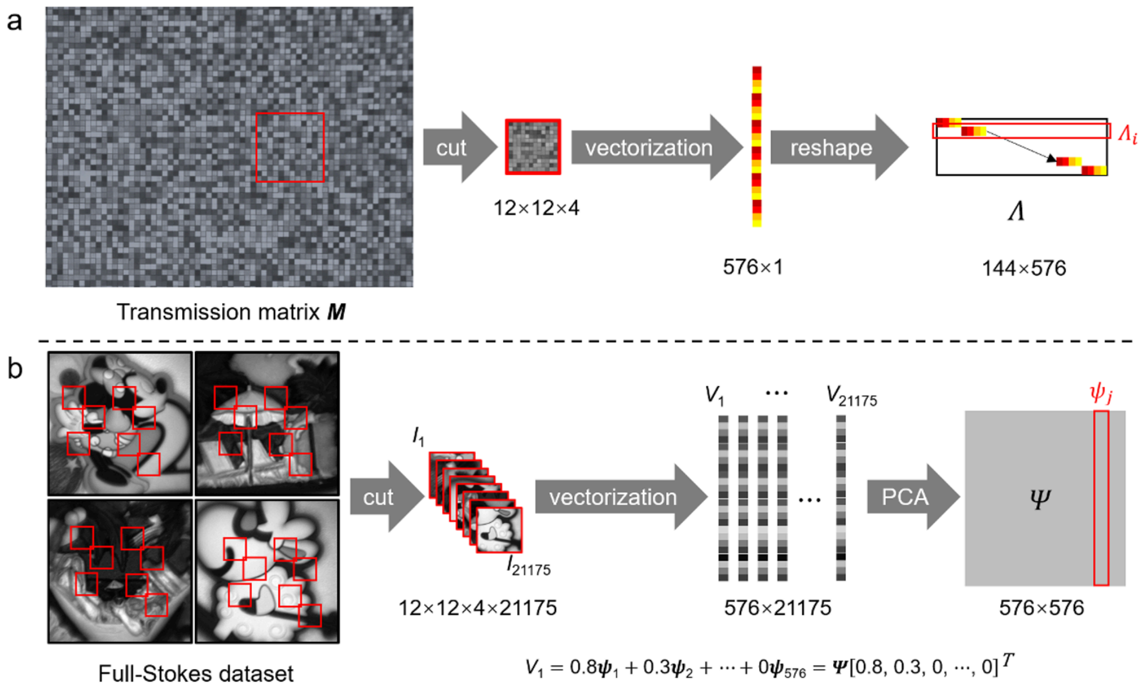

Supplementary Fig. 6 | The pipeline for calculating sampling efficiency $\boldsymbol{\mu}(\boldsymbol{\Lambda}, \boldsymbol{\Psi})$. (a) Calculation process for matrix $\boldsymbol{\Lambda}$. (b) Generation of sparse basis $\boldsymbol{\Psi}$. The size of the corresponding image patch is marked below. There are totally 21,175 image vectors cut from the full-Stokes polarization dataset. Then 576 uncorrelated basis vectors are extracted from these image vectors utilizing the PCA method and combined into a sparse representation matrix $\boldsymbol{\Psi}$ in descending order of weight.

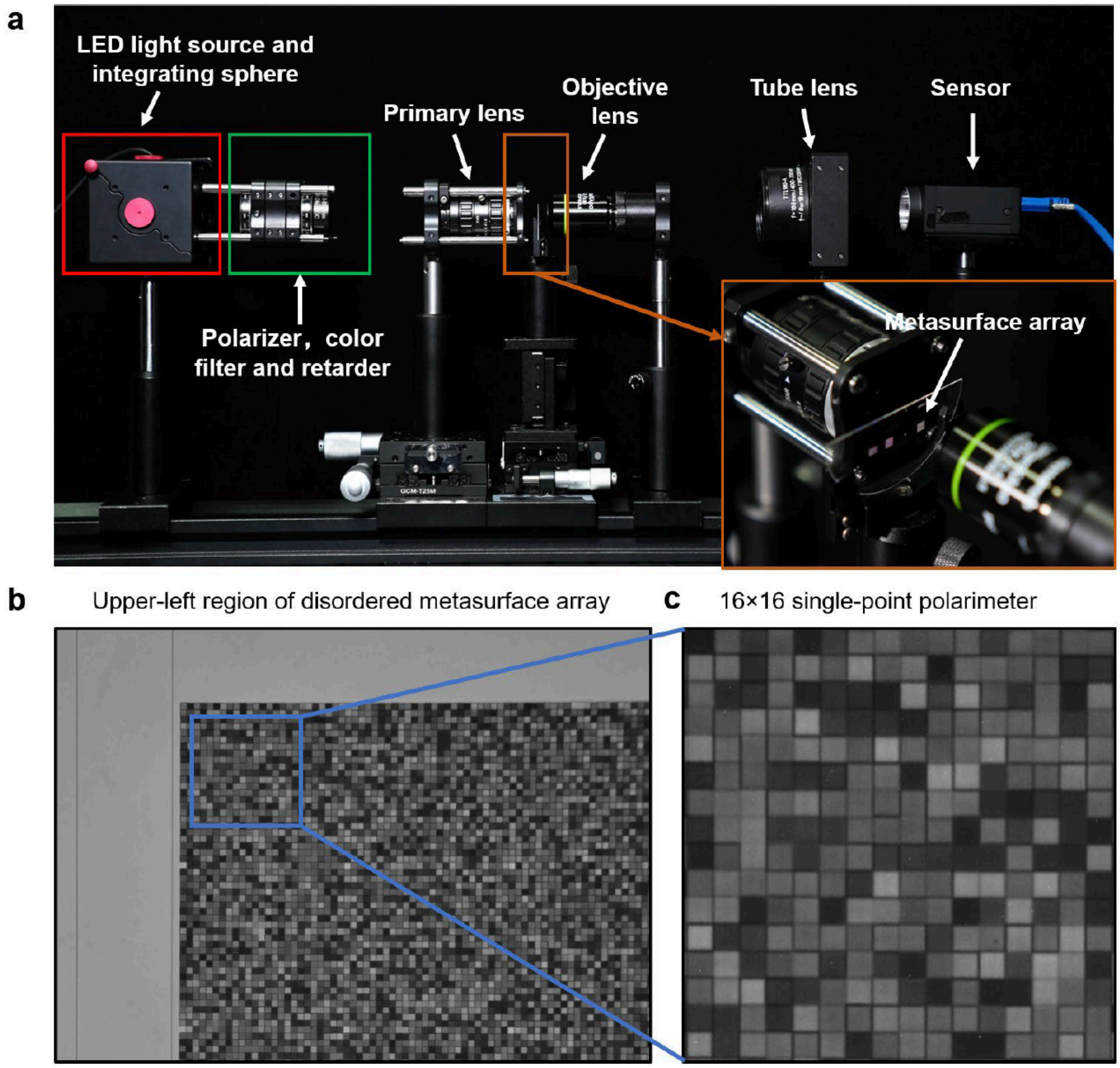

Supplementary Fig. 7 | (a) The optical setup for the polarimetric measurements. The red box is the LED light source and the integrating sphere. The uniform LED light is emitted from the integrating sphere and then enters the system through the linear polarizer, color filter and retarder successively. By rotating the angle of the polarizer and retarder, arbitrary SoP can be generated. The inset shows the disordered metasurface array (shown in orange box). (b-c) In our experiment, we selected a 16×16 subarray located in the upper-left region of the device to conduct the polarization measurement.

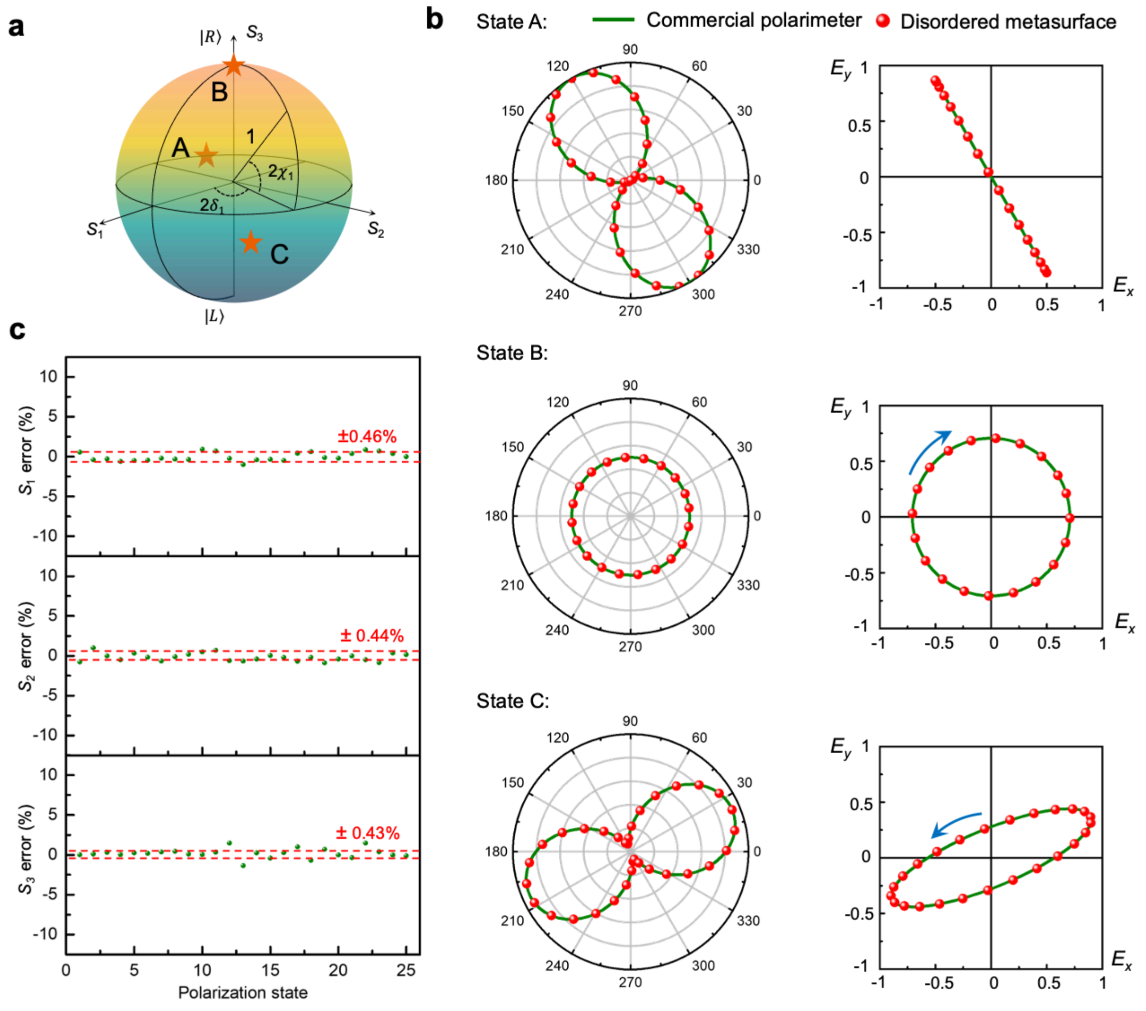

Supplementary Fig. 8 | Full-Stokes polarimetric measurements at the wavelength of 650 nm. (**a**) Three representative SoPs are chosen for polarization measurement. (**b**) Comparison of the SoPs obtained using a commercial polarimeter (green solid lines) and our method (red dots), using polar plots and polarization ellipses. The radius on the polar plot indicates the normalized light intensity. Blue arrows denote the handedness of light. (**c**) The reconstruction errors of Stokes parameters (S_1, S_2, S_3) of 25 arbitrarily selected SoPs on Poincaré sphere.

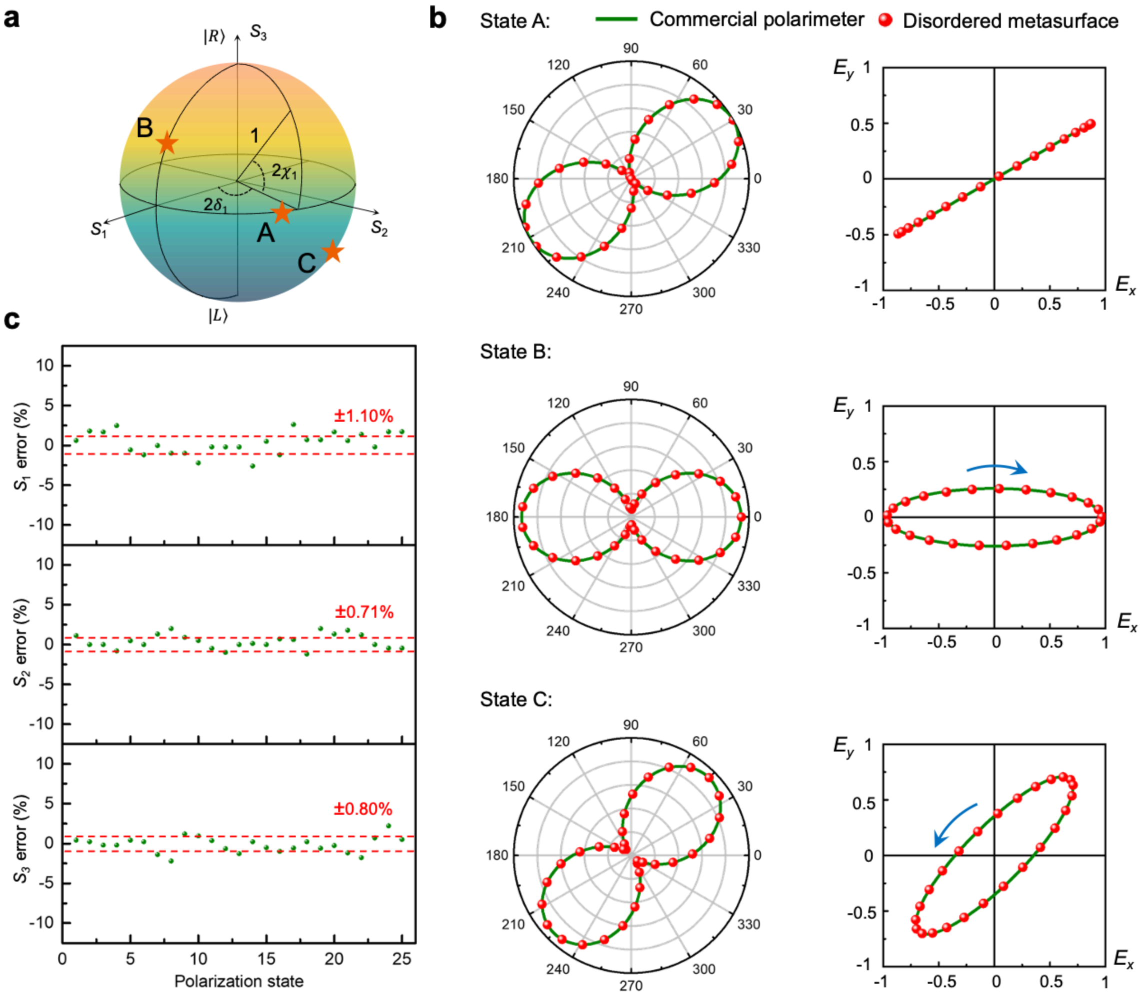

Supplementary Fig. 9 | Full-Stokes polarimetric measurements at the wavelength of 450 nm. (**a**) Three representative SoPs are chosen for polarization measurement. (**b**) Comparison of the SoPs obtained using a commercial polarimeter (green solid lines) and our method (red dots), using polar plots and polarization ellipses. The radius on the polar plot indicates the normalized light intensity. Blue arrows denote the handedness of light. (**c**) The reconstruction errors of Stokes parameters (S_1, S_2, S_3) of 25 arbitrarily selected SoPs on Poincaré sphere.

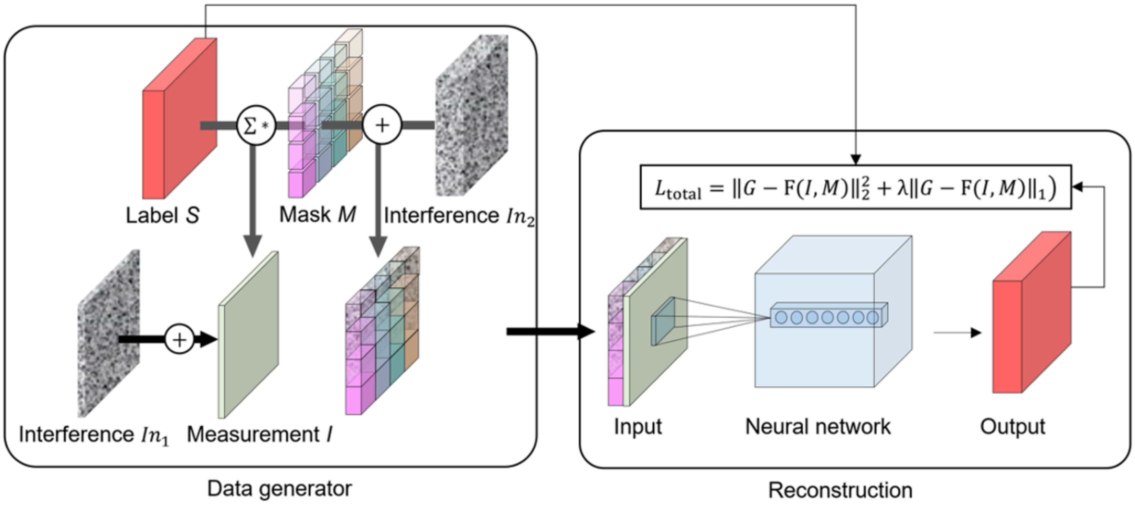

Supplementary Fig. 10 | The framework of the reconstruction algorithm. The reconstruction process is divided into two steps, the polarization data generator and the full convolutional neural network. $\sum *$ represents the forward imaging process, i.e., the label (or the target full-Stokes polarization image) first point-wise multiplies the polarization transmission matrix $\boldsymbol{M}$, then integrates over the polarization dimension. The interference In_1 corresponds to the noise in image system and the interference In_2 is used to simulate the calibration errors in transmission matrix $\boldsymbol{M}$. Through adding these interferences during the training process, the trained neural network could be robust to noise interferences existed in the imaging process.

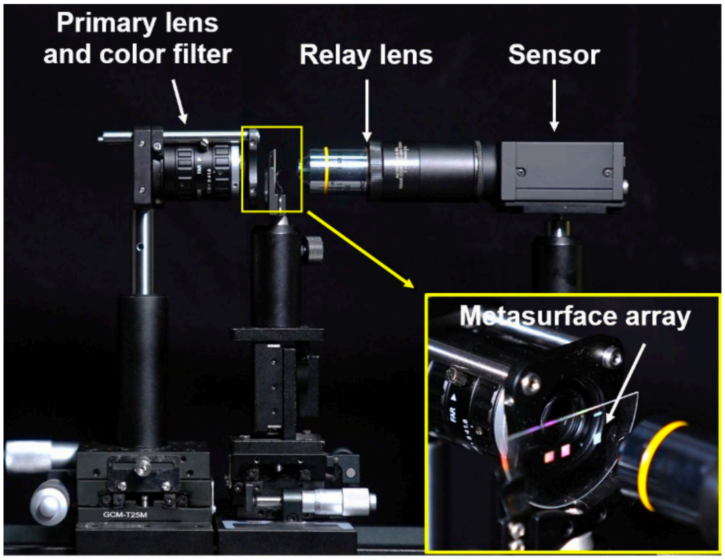

Supplementary Fig. 11 | The optical setup of the proposed polarization imaging system. The inset shows the fabricated disordered metasurface array.

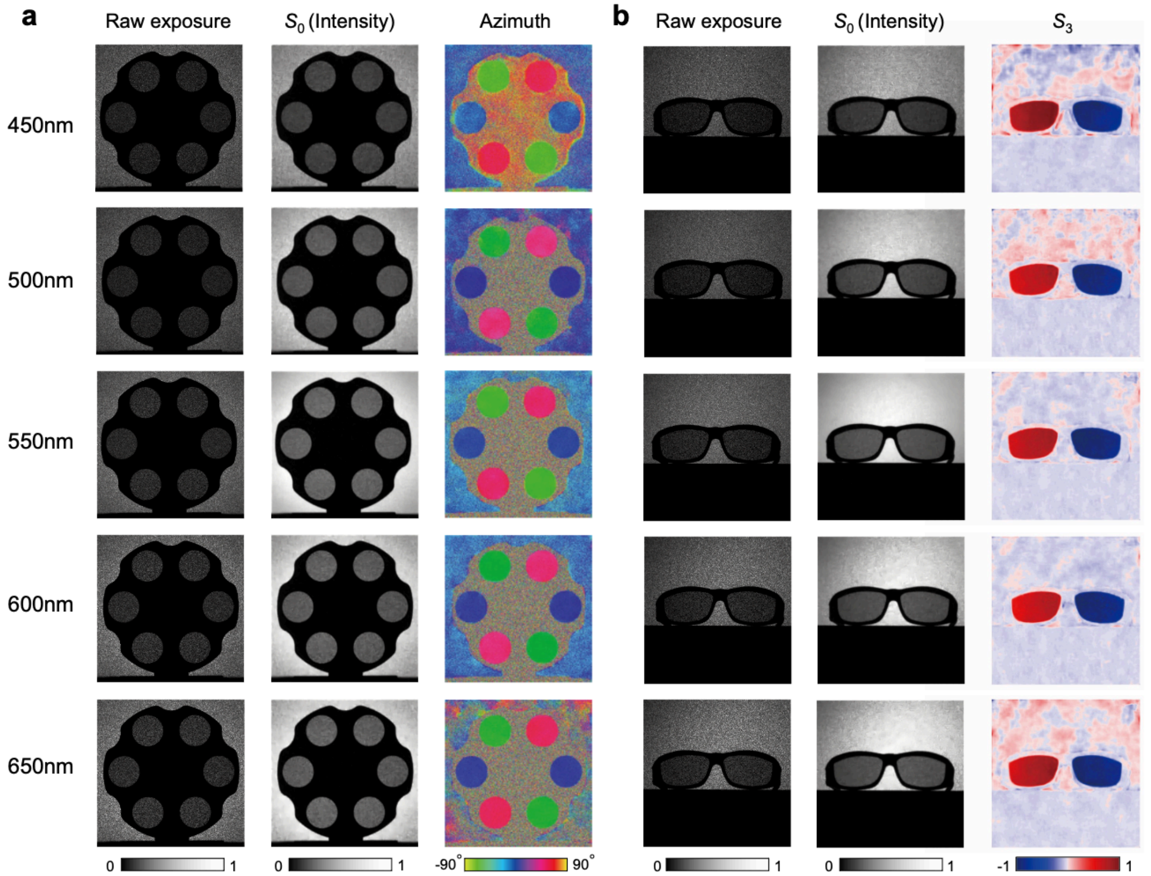

Supplementary Fig. 12 | Polarization imaging at different wavelengths in the visible spectrum. Linear (**a**) and circular (**b**) polarization imaging with the proposed camera. The raw unprocessed exposure, S_0 (corresponding to the traditional monochrome intensity image), the azimuth of the polarization ellipse, and S_3 are shown. Illumination was provided by LED which passes through a 10 nm bandpass filter.

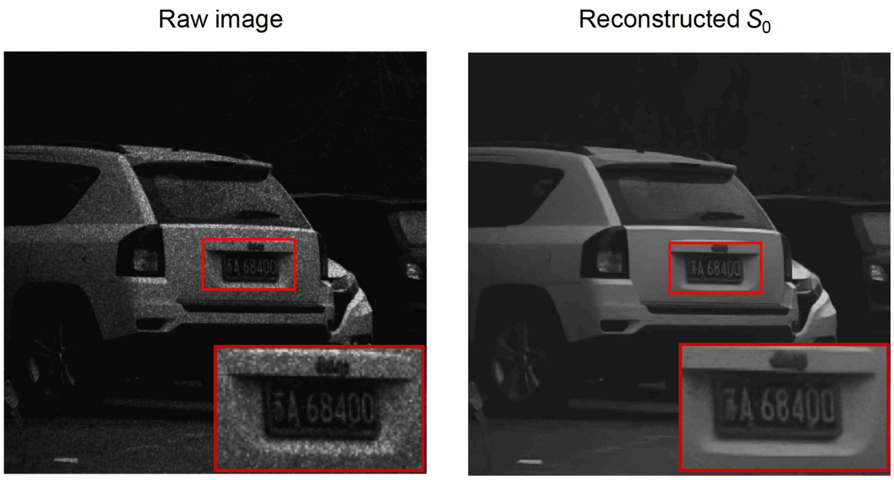

Supplementary Fig. 13 | The reconstructed intensity image S_0 of the pixel-level scene in Fig. 5. The car plate is scaled into the red box. The numbers on car plate are only one or two meta-pixels in width and can still be reconstructed clearly.

Supplementary references

1. Q. B. Fan, M. Z. Liu, C. Zhang, W. Q. Zhu, Y. L. Wang, P. C. Lin, F. Yan, L. Chen, H. J. Lezec, Y. Q. Lu, A. Amit, T. Xu, Independent amplitude control of arbitrary orthogonal states of polarization via dielectric metasurfaces. *Phys. Rev. Lett.* **125**(26), 267402 (2020).
2. J. P. B. Mueller, N. A. Rubin, R. C. Devlin, B. Groever, F. Capasso, Metasurface Polarization Optics: Independent Phase Control of Arbitrary Orthogonal States of Polarization. *Phys. Rev. Lett.* **118**(11) , 113901 (2017).
3. E. Arbabi, S. M. Kamali, A. Arbabi, A. Faraon, Full-Stokes imaging polarimetry using dielectric metasurfaces. *ACS Photon.* **5**(8), 3132-40 (2018).
4. W. L. Hsu, G. Myhre, K. Balakrishnan, N. Brock, M. Ibn-Elhaj, and S. Pau, Full-Stokes imaging polarimeter using an array of elliptical polarizer. *Opt. Express* **22**(3), 3063-3074 (2014)
5. K. A. Bachman, J. J. Peltzer, P. D. Flammer, T. E. Furtak, R. T. Collins, and R. E. Hollingsworth , Spiral plasmonic nanoantennas as circular polarization transmission filters. *Opt. express* **20**(2),1308-19 (2012).
6. A. Fan, T. Xu, G. Teng, X. Wang, Y. Zhang, C. Xu, X. Xu, J. Li, Full-Stokes polarization multispectral images of various stereoscopic objects. *Sci. Data* 10, 328 (2023).
7. D. W. Marquardt, An algorithm for least-squares estimation of nonlinear parameters. *Journal of the society for Industrial and Applied Mathematics* **11**(2), 431-41 (1963).
8. K. Lee, H. Yun, S. E. Mun, G. Y. Lee, J. Sung, and B. Lee, Ultracompact broadband plasmonic polarimeter. *Laser Photon. Rev.* **12**(3), 1700297 (2018).
9. J. P. B. Mueller, K. Leosson, and F. Capasso, Ultracompact metasurface in-line polarimeter. *Optica* **3**(1), 42–47 (2016).
10. A. Espinosa-Soria, F. J. Rodríguez-Fortuño, A. Griol, and A. Martínez, On-chip optimal Stokes nanopolarimetry based on spin–orbit interaction of light. *Nano Lett.* **17**(5), 3139-3144 (2017).
11. A. Basiri, X. Chen, J. Bai, P. Amrollahi, J. Carpenter, Z. Holman, and Y. Yao, Nature-inspired chiral metasurfaces for circular polarization detection and full-Stokes polarimetric measurements. *Light Sci. Appl.* **8**(1), 1-11 (2019).
12. Y. Dai, Y. Zhang, Y. Xie, D. Wang, X. Wang, T. Lei, and X. Yuan, Multifunctional

geometric phase optical element for high-efficiency full Stokes imaging polarimetry. *Photon. Res.* **7**(9), 1066-1074 (2019).

13. J. Bai, C. Wang, X. Chen, A. Basiri, C. Wang, and Y. Yao, Chip-integrated plasmonic flat optics for mid-infrared full-Stokes polarization detection. *Photon. Res.* **7**(9), 1051-1060 (2019).
14. A. Pors, M. G. Nielsen, and S. I. Bozhevolnyi, Plasmonic metagratings for simultaneous determination of Stokes parameters. *Optica* **2**, 716–723 (2015).
15. I. Choi, M. H. Kim, D. Gutierrez, D. S. Jeon, G. Nam, High-quality hyperspectral reconstruction using a spectral prior. *ACM Trans. Graphic* **36**, 104309 (2017)
16. A. Chakrabarti, T. Zickler, Statistics of real-world hyperspectral images. *Proceedings of the IEEE Computer Vision and Pattern Recognition,* 193-200 (2011).
17. F. Yasuma, T. Mitsunaga, D. Iso, S. K. Nayar, Generalized assorted pixel camera: postcapture control of resolution, dynamic range, and spectrum. *IEEE Trans. Image Proc.* **19**(9), 2241-2253 (2010).
18. D. H. Foster, K. Amano, S. M. Nascimento, M. J. Foster, Frequency of metamerism in natural scenes. *J. Opt. Soc. of Am. A* **23**(10), 2359-2372 (2006).
19. S. M. Nascimento, F. P. Ferreira, D. H. Foster, Statistics of spatial cone-excitation ratios in natural scenes. J. Opt. Soc. of Am. A 19(8), 1484-1490 (2002).
20. E. Candès, M. Wakin, S.P.Boyd, Enhancing Sparsity by Reweighted ℓ1 Minimization. J. Fourier Anal. Appl. 14, 877–905 (2008).
