# Supplementary material for: Disordered metasurface enabled single-shot full-Stokes polarization imaging leveraging weak dichroism
Source: Nat Commun. 2023 Nov 7;14:7180. doi: 10.1038/s41467-023-42944-6 (PMC10630513; doi:10.1038/s41467-023-42944-6)
Supplement: Supplementary file 2 — Description of Additional Supplementary Files [file 41467_2023_42944_MOESM2_ESM.pdf]

Description of Additional Supplementary Files:

Supplementary Movie 1: The supplementary movie S1 shows the real-time polarization imaging of a dynamic scene where a rotating filter wheel with six sheets of film polarizer whose axes are arranged radially outward.
